# Lack of serotonin reuptake during brain development alters rostral raphe-prefrontal network formation

**DOI:** 10.3389/fncel.2013.00143

**Published:** 2013-10-04

**Authors:** Josefine S. Witteveen, Anthonieke Middelman, Josephus A. van Hulten, Gerard J. M. Martens, Judith R. Homberg, Sharon M. Kolk

**Affiliations:** ^1^Department of Molecular Animal Physiology, Donders Institute for Brain, Cognition and Behaviour, Radboud University NijmegenNijmegen, Netherlands; ^2^Department of Cognitive Neuroscience, Donders Institute for Brain, Cognition and Behaviour, Radboud University Nijmegen Medical CentreNijmegen, Netherlands

**Keywords:** explant assays, prefrontal cortex, serotonin transporter, microdissection, axon guidance, depression, SERT, autism

## Abstract

Besides its “classical” neurotransmitter function, serotonin (5-HT) has been found to also act as a neurodevelopmental signal. During development, the 5-HT projection system, besides an external placental source, represents one of the earliest neurotransmitter systems to innervate the brain. One of the targets of the 5-HT projection system, originating in the brainstem raphe nuclei, is the medial prefrontal cortex (mPFC), an area involved in higher cognitive functions and important in the etiology of many neurodevelopmental disorders. Little is known, however, about the exact role of 5-HT and its signaling molecules in the formation of the raphe-prefrontal network. Using explant essays, we here studied the role of the 5-HT transporter (5-HTT), an important modulator of the 5-HT signal, in rostral raphe-prefrontal network formation. We found that the chemotrophic nature of the interaction between the origin (rostral raphe cluster) and a target (mPFC) of the 5-HT projection system was affected in rats lacking the 5-HTT (5-HTT^−/−^). While 5-HTT deficiency did not affect the dorsal raphe 5-HT-positive outgrowing neurites, the median raphe 5-HT neurites switched from a strong repulsive to an attractive interaction when co-cultured with the mPFC. Furthermore, the fasciculation of the mPFC outgrowing neurites was dependent on the amount of 5-HTT. In the mPFC of 5-HTT^−/−^ pups, we observed clear differences in 5-HT innervation and the identity of a class of projection neurons of the mPFC. In the absence of the 5-HTT, the 5-HT innervation in all subareas of the early postnatal mPFC increased dramatically and the number of Satb2-positive callosal projection neurons was decreased. Together, these results suggest a 5-HTT dependency during early development of these brain areas and in the formation of the raphe-prefrontal network. The tremendous complexity of the 5-HT projection system and its role in several neurodevelopmental disorders highlights the need for further research in this largely unexplored area.

## Introduction

It has become increasingly clear that several “classical” neurotransmitters, such as serotonin (5-HT), additionally act as neurodevelopmental signals to direct the assembly of the developing brain (Lauder, 1990; Whitaker-Azmitia et al., 1996; Buznikov et al., 2001; Sodhi and Sanders-Bush, 2004; Cunningham et al., 2005; Riccio et al., 2009; Souza and Tropepe, 2011; Bonnin and Levitt, 2012; Migliarini et al., 2012). Even before the raphe-derived neurites start extending, there is an external placental source of 5-HT (Bonnin et al., 2011). Furthermore, 5-HT signaling molecules such as enzymes responsible for 5-HT synthesis and breakdown, 5-HT receptors and the 5-HT transporter (5-HTT) are already expressed in the brain before 5-HT neurons are born (Bruning et al., 1997; Zhou et al., 2000; Cote et al., 2007; Bonnin et al., 2011; Bonnin and Levitt, 2012). The role of 5-HT and its signaling molecules during development is especially important in the light of recent discussions on the effect of serotonin-reuptake inhibitors (SSRIs) during pregnancy (Vitalis et al., 2007; Alwan and Friedman, 2009; Oberlander et al., 2009; Gentile and Galbally, 2011; Simpson et al., 2011). SSRIs given to the pregnant mother to treat depression, will increase the extracellular 5-HT in not only the mother but also in the brains of the unborn child (Rampono et al., 2004; Gentile and Galbally, 2011). These children acquire an increased risk to develop reduced somatosensory responses (Oberlander et al., 2009) and/or psychomotor control (Casper et al., 2011), and appear to have a higher risk to develop autism-like symptoms (Croen et al., 2011).

The 5-HT projection system is one of the earliest neurotransmitter systems to develop and send out its projections to distant targets (Whitaker-Azmitia, 2001; Homberg et al., 2010). Specifically, the 5-HT neurons located in the rostral raphe cluster extend profuse axon tracts into the fore- and midbrain (Gaspar et al., 2003; Bang et al., 2012). A distant target of the ascending 5-HT projection system within the forebrain is the medial prefrontal cortex (mPFC) (Del Cid-Pellitero and Garzon, 2011; Waselus et al., 2011). The mPFC is the seat of our highest cognitive abilities and known to be involved in attentional processes, working memory and behavioral flexibility (Miller and Cohen, 2001; Heidbreder and Groenewegen, 2003). In rodents, the developing 5-HT-positive fibers reach the mPFC around embryonic day 16–17 (E16 in mouse and E17 in rats), where they initially innervate the marginal zone and the subplate, before massively innervating the cortical plate proper (Janusonis et al., 2004). The 5-HT fibers, found within the marginal zone of the mPFC, are thought to contact Cajal-Retzius (CR) cells, cortical layer I cells secreting the glycoprotein reelin crucial for the correct layering of the cortex (Janusonis et al., 2004; Leemhuis et al., 2010). These CR cells express 5-HT_1A_ and 5-HT_3A_ receptors (Janusonis et al., 2004; Chameau et al., 2009; Vucurovic et al., 2010) and differences in 5-HT input onto the latter could result in an altered reelin release, cortical layering and ultimately, PFC-mediated cognitive functioning. Indeed, altered 5-HT innervations of the mPFC have been implicated in the etiology of neurodevelopmental disorders such as schizophrenia, autism spectrum disorders (ASD) and intellectual disability (Gurevich and Joyce, 1997; Chugani et al., 1999; Whitaker-Azmitia, 2001, 2005; Canli et al., 2005; Canli and Lesch, 2007; Robbins and Arnsten, 2009; Costa et al., 2012; Mann, 2013).

There are indications that 5-HT acts as a soluble cue and modulates the response of targeting axons to guidance cues (Petit et al., 2005; Bonnin et al., 2007). Due to the important role of 5-HT in neurodevelopment, factors that influence 5-HT signaling may also have profound effects on the correct development of the brain. The presynaptically located 5-HTT is the primary regulator of 5-HT signaling, terminating the 5-HT signal by allowing reuptake for recycling or degradation (Homberg et al., 2007a, 2010; Neumann et al., 2011). Apart from being expressed in 5-HT neurons, the 5-HTT is also transiently expressed in non-aminergic neurons, belonging to many topographically distinct brain areas (Lebrand et al., 1998; Zhou et al., 2000; Narboux-Neme et al., 2008; Kalueff et al., 2010). In humans, the 5-HTT gene-linked polymorphic region (5-HTTLPR), composed of a short and a long version (Canli and Lesch, 2007; Neumann et al., 2011; Haddley et al., 2012), affects 5-HTT expression and function. The short (s) variant has been associated with robust neurodevelopmental changes in corticolimbic structures, and increased risk for depression in the context of stress (Canli and Lesch, 2007). The 5-HTT^−/−^ rat model is also known to display anxiety- and depression-related responses to stressors (Homberg et al., 2007b; Kalueff et al., 2010). Extracellular levels of 5-HT are increased throughout the brain of the 5-HTT^−/−^ rodent, and affect 5-HT receptor expression, where the 5-HT_1A_ is known to be down-regulated in both 5-HTT^−/−^ rodents and s-variant carriers (David et al., 2005; Riccio et al., 2009; Kalueff et al., 2010). Also, due to 5-HTT deficiency, increased activity at the 5-HT_6_ receptor affects proper cortical cytoarchitecture and interneuron migration (Riccio et al., 2009, 2011). The mechanisms by which a reduced 5-HTT function in humans, or reduction/deficiency of 5-HTT in rodents, and consequent increased 5-HT levels, affects areal maturation, guidance and network formation are still not fully understood.

Here we report the results of our study of the chemotropic nature of the interaction between the origin (rostral raphe cluster) and a target (mPFC) of the 5-HT projection system using the 5-HTT knockout rat model. Additionally, we have examined the ability of the outgrowing neurites to form fascicles, and whether differences in fasciculation could be due to 5-HTT deficiency. Moreover, in order to determine whether the early lack of the 5-HTT also affected the maturation of the 5-HT raphe-mPFC projection system, we examined the 5-HT innervation within various subareas of the mPFC in 5-HTT^−/−^ and 5-HTT^+/+^ pups. Using the transcription factor Satb2 (special AT-rich sequence binding protein 2) as a marker for callosal projection neurons in cortical layers II-VI, we analyzed the number of Satb2-positive neurons in 5-HTT-deficient pups.

## Materials and methods

### Animals

All animal use and care were performed in accordance with the institutional and national guidelines and regulations of the Committee for Animal Experiments of the Radboud University Nijmegen, The Netherlands. All animal experiments conformed to the relevant regulatory standards. The 5-HTT mutant rats (Slc6a4^1Hubr^) were generated in a Wistar background by target-selected ENU-induced mutagenesis (for detailed description, see Smits et al., 2006). Timed-pregnant rats were individually housed in macrolon cages in a temperature- and humidity-controlled room (21 ± 1°C and 60% relative humidity, respectively). The rats had *ad-libitum* access to food and water and a normal light-dark cycle was maintained. Timed-pregnant rats were sacrificed by means of CO_2_/O_2_. The morning on which a vaginal plug was detected is considered E0.5. Genotyping of the embryos and pups was performed by KBioscience (Hoddesdon, United Kingdom).

### Explant cultures

**Figure 1:**
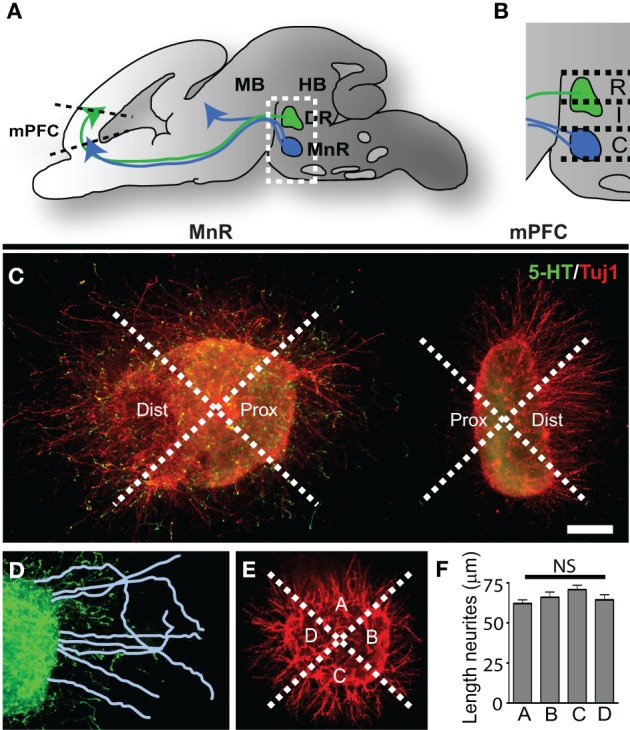
**Three-dimensional collagen co-cultures of explants taken from the mPFC, DR, and MnR show trophic responses. (A)** Schematic of an embryonic brain showing the position of the 5-HT-positive rostral cluster of raphe nuclei projecting to the mid- and forebrain. The dorsal raphe nucleus (DR) projects to forebrain regions (green arrow) including the prefrontal cortex (mPFC). The median raphe nucleus (MnR) projects (blue arrows) to fore-and midbrain regions. **(B)** Enlargement of the boxed area in **(A)**. The rostral (R) and intermediate (I) subarea correspond to the DR and the caudal (C) subarea corresponds to the MnR. **(C)** Example of a 5-HTT^+/−^ caudal subarea (MnR) co-cultured with mPFC, divided in proximal and distal quadrants and stained for 5-HT (5-HT neurites, green) and Tuj1 (β-III tubulin, all outgrowing neurites, red). **(D)** In the proximal (and distal, not shown) quadrants the neurites are traced and measured. **(E)** Control explants were cultured separately and neurite outgrowth was measured in the 4 quadrants (example of WT mPFC). **(F)** The average length of the neurites in quadrants A, B, C, or D showed no significant (NS) difference. HB, hindbrain; MB, midbrain. Scale bar represents 80 μm.

Three-dimensional collagen matrix explant assays were performed as described previously (Kolk et al., 2009). Embryonic day 16.5 (E16.5) rat embryos were collected in ice-cold L15 medium (Leibovitz with L-glutamine, PAA, Austria) and brains were rapidly dissected. Explants (<300 μm) were microdissected from (1) the rostral cluster of raphe nuclei, in a rostral-to-caudal direction dividing it in a rostral, intermediate and caudal subarea, bisected along the midline; and (2) the mPFC. Rostral and intermediate subareas correspond to the dorsal raphe nucleus, and the caudal subarea corresponds to the median raphe nucleus (MnR; Figures 1A,B; Supplemental Figure 1A). The explants were collected in ice-cold L15 medium containing 10% fetal calf serum (FCS).

Combinations of the various raphe subareas and the mPFC were embedded in close proximity (~300 μm apart) in a collagen matrix (10% 10X MEM, Invitrogen; and 10% NaHCO_3_ in diluted rat tail collagen, Invitrogen) in four-well culture dishes (Nunclon surface, Nunc, ThermoScientific). As controls, the various raphe subareas and the mPFC explants were cultured individually to check for their radial growth. Explants were cultured in growth medium (DMEM-F12 with 10% glutamine and antibiotics, 6% 1,7M glucose, and 10% FCS) in a humidified incubator at 37°C with 5% CO_2_ for 4 days. Growth medium was renewed after 24 h. For each of the combinations of co-cultures mentioned above, at least four independent experiments were performed.

### Immunohistochemistry

Brains were rapidly dissected from E16.5 embryos and postnatal day 6 (P6) pups, fixed by immersion for 90 min. in 4% paraformaldehyde (PFA) in phosphate-buffered saline (PBS), washed in PBS and cryoprotected in 30% sucrose in PBS. Brains were frozen in M-1 embedding matrix (Thermo Fisher Scientific) on dry ice in a plastic cup and stored at −80^°^C. Cryostat sections were cut at 16 μm, mounted on Superfrost Plus slides (Thermo Fisher Scientific), air-dried, and stored desiccated at −20^°^C.

Cryosections were stained immunohistochemically as described previously (Kolk et al., 2006). Rabbit anti-5-HT (Sigma, 1:5000) and mouse anti-Satb2 (Abcam, 1:500) were diluted in blocking buffer (BB, 1.7% normal donkey serum, 1.7% normal goat serum, 1.7% normal horse serum, 1% BSA, 1% glycine, 0.1% lysine, 0.4% Triton X-100, in PBS) and incubated overnight at 4°C. Sections were incubated in species-specific Alexa-conjugated secondary antibody (Molecular Probes) generated in goat and diluted 1:500 in BB for 30 min. at RT. After washing in PBS, sections were counterstained with fluorescent Nissl stain (NeuroTrace; Invitrogen; 1:500), washed extensively in PBS, and embedded in 90% glycerol. Antibody specificity was tested by omitting the primary antibody resulting in no positive signal (negative control) and careful comparison of immuno-positive brain areas with the areas described before (Riccio et al., 2009; Balamotis et al., 2012) (positive control). The nomenclature to describe 5-HT-positive cells and fibers within various brain areas is as described by (Abrams et al., 2004; Kolk et al., 2009).

The explants in their collagen matrix were quickly washed in PBS, fixed in buffered 4% PFA for 1.5 h, and washed extensively o/n at 4°C before performing immunocytochemistry. Explants were incubated in BB for 6–8 hr at room temperature (RT). The explants were incubated with primary antibody diluted in BB o/n at 4°C. Rabbit anti-5-HT (Sigma, 1:5000) and mouse anti-Tuj1 (β-III tubulin, Covance, 1:1000) were used to visualize 5-HT or all outgrowing neurites, respectively. On the second day, explants were washed 4 times for a total of 4–5 h at RT. They were then incubated with species-specific Alexa-conjugated secondary antibody (Molecular Probes) generated in goat and diluted 1:500 in BB for 1 h at RT. After washing extensively in PBS o/n at 4°C, the explants were embedded (Prolong Gold, antifade reagent, Invitrogen). For visualization, a Leica DMRA Fluorescence microscope with DFC340FX camera and LASAF software was used.

### Data analysis

All data analysis was performed in a double-blind fashion. For quantification of the explants assays, the explants were divided in a proximal and distal quadrant of which images were captured as described by (Kolk et al., 2009). The length of the 20 longest neurites was measured in both the proximal and distal quadrants of the culture using Neuron J (Image J plug-in) with an average of 5 explants per condition. The average value of length of each explant in both proximal and distal quadrants was used to determine the proximal/distal ratio (P/D ratio) per explant (Kolk et al., 2009). The number, width, and length of the neurite fascicles were analyzed in the proximal and distal quadrants of the culture using NeuronJ by tracing across and along the fascicle.

For assessing 5-HT fiber length and number of Satb2-positive neurons in the various subareas of the mPFC of 5-HTT^+/+^, 5-HTT^+/−^ and 5-HTT^−/−^ rats, three to five pups of each genotype of at least three independent litters were analyzed and two to four well-spaced (120 μm) sections at the same neuroanatomical level were imaged. A 0.1-mm-wide rectangle spanning the cerebral wall was placed over the center of the subarea (either infralimbic, IL, prelimbic, PL or cingulate cortex, Cg) of the mPFC. The overall cortical width of a subarea was divided into 10 equal bins [bin 1 within the deep cortical zone and bin 10 within the presumptive layer I] within this rectangle, and 5-HT-positive fiber length or Satb2/Nissl-positive neuron number was measured within each bin using ImageJ software (NIH, Bethesda, USA). Data were normalized to total length or number per square micrometer and averaged for each pup. To better visualize and compare 5-HT innervation of wild-type and mutant mPFC, reconstructions of the individual fibers were obtained using NeuronJ from two to three consecutive sections, bilaterally. Data were statistically analyzed by one-way ANOVA (α = 5%) and expressed as means ± SEM.

## Results

### Presence of 5-HTT during early development modulates rostral raphe-mPFC directional responses *in vitro*

The rostral cluster of raphe nuclei forms projections toward their targets in the fore- and midbrain (Dahlstrom and Fuxe, 1964; Van Bockstaele et al., 1993; Waselus et al., 2011; Bang et al., 2012). One of the targets within the forebrain is the mPFC (Wilson and Molliver, 1991; Verney et al., 2002; Del Cid-Pellitero and Garzon, 2011; Puig and Gulledge, 2011). The 5-HT projections are guided along the way to their target by various cues, either soluble or membrane-bound, as they develop (Petit et al., 2005; Anitha et al., 2008; Lee et al., 2010).

To identify the chemotropic nature of the interaction between the rostral cluster of raphe nuclei and the mPFC and to evaluate possible changes in the 5-HTT knockout model, we performed three-dimensional collagen co-cultures of the rostral cluster of raphe nuclei and the mPFC (Figure 1; Supplemental Figures 1A,B,D). Brain areas were microdissected from E16.5 5-HTT^+/+^, 5-HTT^−/−^ and 5-HTT^+/−^ embryonic brains (Supplemental Figure 1A). Explants were taken from the rostral cluster of raphe nuclei and were divided into three subareas; rostral, intermediate and caudal (Figures 1A,B; Supplemental Figure 1A). The rostral and intermediate subareas correspond to the dorsal raphe nucleus (DR) which mainly projects to the forebrain, including the mPFC (Van Bockstaele et al., 1993; Waselus et al., 2011). The caudal subarea corresponds to the median raphe nucleus (MnR) which innervates both the fore- and midbrain (Figures 1A,B; Supplemental Figures 1A–C) (Puig and Gulledge, 2011). The explants from the mPFC were co-cultured with one of the subareas of the raphe in a collagen hill for 4 days (Figure 1; Supplemental Figures 1A,B,D). After 4 days, the explants were fixed and immunostained for 5-HT and Tuj1 (β-III tubulin, a marker for outgrowing neurites). To measure the extent of attraction or repulsion of outgrowing neurites, revealing the chemotrophic nature of the interaction between the two areas, the explants were divided into a proximal and a distal quadrant, with the proximal quadrant facing the co-cultured explant (Figure 1C). Within the proximal and distal quadrant the lengths of the longest neurites were measured and averaged (Figure 1D). The average length of the neurites on the proximal site was then divided by the average length of the distal site neurites giving the proximal/distal-ratio (P/D ratio). A P/D ratio above 1 indicates an attractive interaction, whereas a P/D ratio less than 1 denotes repulsion (Pasterkamp et al., 2003; Kolk et al., 2009; Fenstermaker et al., 2010). As a control, explants of the various brain areas were cultured individually and divided into four quadrants (Figure 1E). The lengths of the longest neurites were measured in each quadrant and statistical analysis revealed no significant differences between the 4 quadrants, indicating a radial neurite outgrowth when cultured individually (Figure 1F).

**Figure 2:**
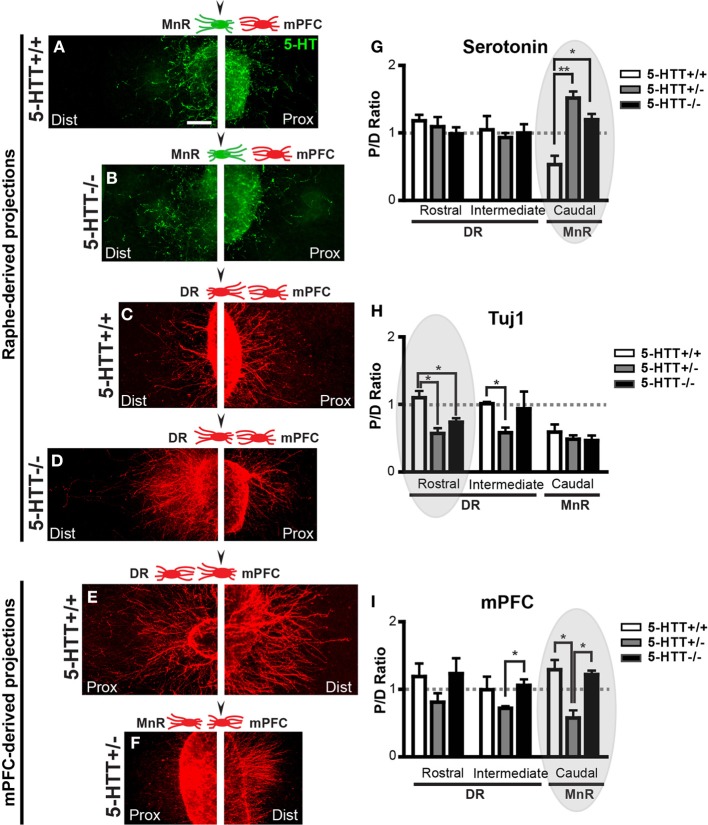
**The chemotrophic nature of the interaction between the mPFC and the DR and MnR depends on 5-HTT during development. (A-F)** High magnification photographs of proximal and distal quadrants of the raphe (positive for 5-HT, green and Tuj1, red) and the mPFC (Tuj1, red). Arrowhead in schematic representations indicate the example given. **(G,H)** Quantification of the length of 5-HT-positive and Tuj1-positive neurites in the proximal and distal quadrants of the DR and the MnR, co-cultured with mPFC. Graphs show average P/D ratios ± SEM. **(I)** Quantification of the length of Tuj1-positive neurites in the proximal and distal quadrants of the mPFC co-cultured with either the DR or MnR. Graph shows average P/D ratios ± SEM. One-Way ANOVA (α = 0.05), ^*^p < 0.05, ^**^p < 0.01. Gray ovals in G-I indicate the example given on the left. Dist, distal quadrant; DR, dorsal raphe nucleus; mPFC, medial prefrontal cortex; MnR, median raphe nucleus; Prox, proximal quadrant.

Figures 2A–F show examples of the proximal and distal side of explants of subareas of the rostral raphe and the mPFC co-cultured together (arrowheads above the schematic indicate the displayed explant). P/D ratios of the 5-HT neurite outgrowth and the overall neurite outgrowth (Tuj1-positive) of suareas of the raphe co-cultured with the mPFC (examples in Figures 2A–D) were calculated and depicted in Figures 2G,H. The P/D ratios of 5-HT neurite outgrowth of the DR (rostral and intermediate subarea) indicated little attraction toward the mPFC (Figure 2G). Lack of 5-HTT had no effect on the targeted neurite outgrowth from the DR (Figure 2G). The P/D ratios of the 5-HT neurite outgrowth of the MnR (caudal subarea) showed significant differences due to 5-HTT deficiency (Figures 2A,B,G). In the wild-type situation a repulsive interaction toward the mPFC was observed (P/D ratio, 0.53; Figures 2A,G). However, the reduction or lack of the 5-HTT caused a significant attractive interaction (P/D ratios, 1.52 and 1.20, respectively (resp.); *p* = 0.0018 and 0.033, resp.; Figures 2B,G). Since the fibers are no longer repulsed, we can speculate that an increased number of 5-HT fibers may now target the mPFC. The observed switch to an attractive interaction was not found in the overall neurite outgrowth (Tuj1) from the MnR, here the interaction remained repulsive (P/D ratios, 0.59, 0.49, 0.47 for HTT^+/+^, 5-HTT^+/−^, and 5-HTT^−/−^, resp.; Figure 2H). In the wild-type situation, the overall neurite outgrowth of the DR (rostral and intermediate explants) seemed to be slightly attracted by the mPFC (P/D ratio, 1.10 and 1.01; Figures 2C,H). However, the 5-HTT^+/−^ situation resulted in a switch to significant repulsion, although with the complete lack of 5-HTT this repulsion became less obvious (P/D ratios, 0.57 and 0.74, resp.; *p* = 0.012 and 0.022, resp.), especially in the more caudal (intermediate subarea) part of the DR (P/D ratios, 0.58 and 0.94, resp.; *p* = 0.022 between HTT^+/+^and 5-HTT^+/−^; Figures 2D,H).

Figures 2D,I show the P/D ratios of overall neurite outgrowth from the mPFC which was co-cultured with either the rostral, intermediate, or caudal subarea of the raphe. The mPFC neurite outgrowth in the wild-type and 5-HTT^−/−^ situation was attractive toward all subareas of the rostral raphe (P/D ratios, 1.19, 1.01 and 1.29, resp.; Figures 2E,F,I). However, for the 5-HTT^+/−^ explants this interaction of the mPFC with all subareas of the rostral raphe switched to repulsive (P/D ratios, 0.81, 0.72 and 0.58, resp.). This phenomenon was most prominent when the mPFC was co-cultured with the MnR (caudal subarea) as shown in Figures 2E,F,I (*p* = 0.017 between HTT^+/+^and 5-HTT^+/−^). In absence of the 5-HTT, the P/D ratios were comparable with the wild type situation (P/D ratios, 1.23, 1.06, and 1.22, resp.; Figure 2I).

Taken together, these data show that the outgrowing neurites of the DR/MnR and from the mPFC, show directional responses when cultured together. The nature of this response is different for DR compared to MnR, and is affected by the level of 5-HTT expression.

### Presence of 5-HTT during early development moderates fasciculation of outgrowing mpfc neurites toward rostral raphe

During the formation of neuronal projection systems, outgrowing axons are guided to their distant targets by a variety of guidance cues (Tessier-Lavigne and Goodman, 1996; Dickson, 2002). Most axons grow alongside other axons for much of their lengths as pioneer axons create the first scaffold for the different projection pathways. Subsequent axons may associate in specific bundles or fascicles, and grow alongside this scaffold in order to reach their proper targets (Van Vactor, 1998; Jaworski and Tessier-Lavigne, 2012). The process of fasciculation of axons requires internal membrane-bound cues, such as members of the neuronal cell adhesion molecule (NCAM) or semaphorin family (Barry et al., 2010). However, soluble guidance cues secreted from (intermediate) targets also modulate fasciculation (Jaworski and Tessier-Lavigne, 2012).

**Figure 3:**
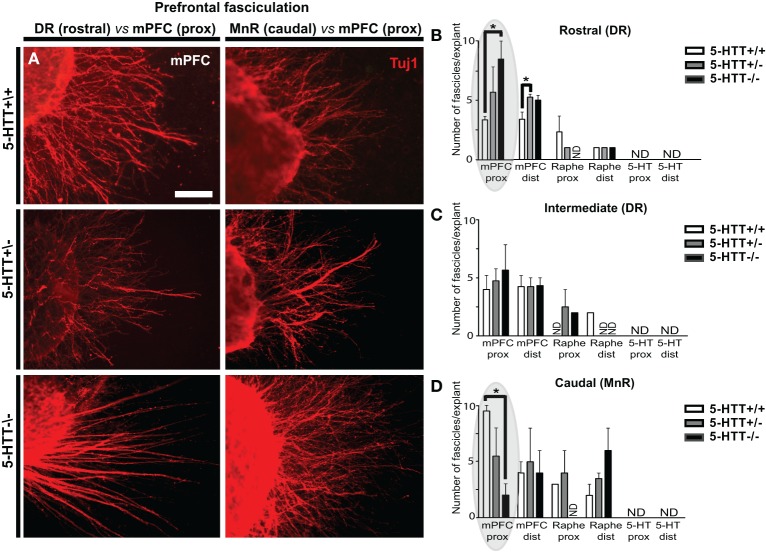
**The amount of 5-HTT during development influences mPFC fasciculation. (A)** Examples of differences in number of fascicles per explant in WT, 5-HTT^+/−^ and 5-HTT^−/−^ mPFC (proximal quadrant) co-cultured with the rostral (DR, left panels) or caudal subarea (MnR, right panels). Scale bar represents 50 μm. **(B–D)** Quantification of the number of mPFC fascicles (>5 μm) per explant, for the different subareas and genotypes in both the proximal (prox) and distal (dist) quadrant. Gray ovals in **(B,D)** indicate the example given on the left. Graphs show average number of fascicles (>5 μm) per explant ± SEM. One-Way ANOVA (α = 0.05), ^*^p < 0.05.

When examining the outgrowing neurites of the explants from the various subareas and genotypes, we noticed differences in the number of outgrowing fascicles, especially from the mPFC (Figure 3; Supplemental Figure 2A). Therefore, we first determined in how many of the explants (proximal quadrant) of the different subareas (both raphe as well as mPFC), fascicles with a minimum width of 5 μm, were formed (Supplemental Figures 2C–E). It became obvious that most of the mPFC explants exhibited fasciculation. Considering exclusively the 5-HT-positive neurites, no fascicles were formed in explants either from the DR or the MnR (Supplemental Figures 2C–F). Lack of 5-HTT did not affect this deficient 5-HT fasciculation. However, in some cases, the 5-HT-positive neurites did grow alongside Tuj1-positive fascicles (Supplemental Figure 1D). In most explants of the mPFC, fascicles were formed, although differences were found when co-cultured with the rostral raphe subareas and across genotypes (Supplemental Figures 2C–E). For example, the percentage of mPFC explants co-cultured with the rostral subarea (DR) showing fascicles was increased in 5-HTT^+/−^ and 5-HTT^−/−^ as compared to the 5-HTT^+/+^ mPFC (Supplemental Figure 2C). All mPFC explants of wild-type and 5-HTT^−/−^ animals co-cultured with the intermediate (DR) and caudal subarea (MnR) had formed fascicles. Notably, the 5-HTT^+/−^ situation resulted in a reduced number of mPFC explants with fascicles co-cultured with the intermediate (DR) and caudal subarea (MnR) (Supplemental Figures 2D,E).

Furthermore, we noticed that the number of fascicles formed per explant varied and depended on the genotype. We therefore quantified the average number of fascicles per mPFC explant. Figure 3A shows the number of fascicles in wild-type (upper panels) as compared to 5-HTT^+/−^ (middle panels) and 5-HTT^−/−^ (lower panels) in the proximal quadrants of mPFC explants co-cultured with either the DR (left panels) or the MnR (right panels). Lack of 5-HTT resulted in a significant increase in the number of fascicles in the proximal quadrant of the mPFC co-cultured with the rostral subarea (DR) of the raphe (average number is 3.33, 6.25 and 7.33 fascicles for HTT^+/+^, 5-HTT^+/−^, and 5-HTT^−/−^, resp.; *p* = 0.023; Figures 3A–D, left) and a significant decrease when co-cultured with the caudal subarea (MnR, Figures 3A,D, right; average number is 9.50, 4.33, and 2.67 fascicles for HTT^+/+^, 5-HTT^+/−^, and 5-HTT^−/−^, resp.; *p* = 0.022). An increase in the number of fascicles per mPFC explant was also observed in the distal quadrant of the mPFC that was co-cultured with the rostral part of the DR upon (partial) lack of the 5-HTT (average number is 3.40, 4.40, and 5.40 fascicles for HTT^+/+^, 5-HTT^+/−^, and 5-HTT^−/−^, resp.; *p* = 0.036; Figure 3B and data not shown).

The length and width of a fascicle may provide information about the number of neurites bundled together, the nature of internal membrane-bound cues, and the nature of (soluble) environmental cues. For example, a repulsive interaction between axon and environment may favor fasciculation by channeling axons in a common path (Van Vactor, 1998; Jaworski and Tessier-Lavigne, 2012).

**Figure 4:**
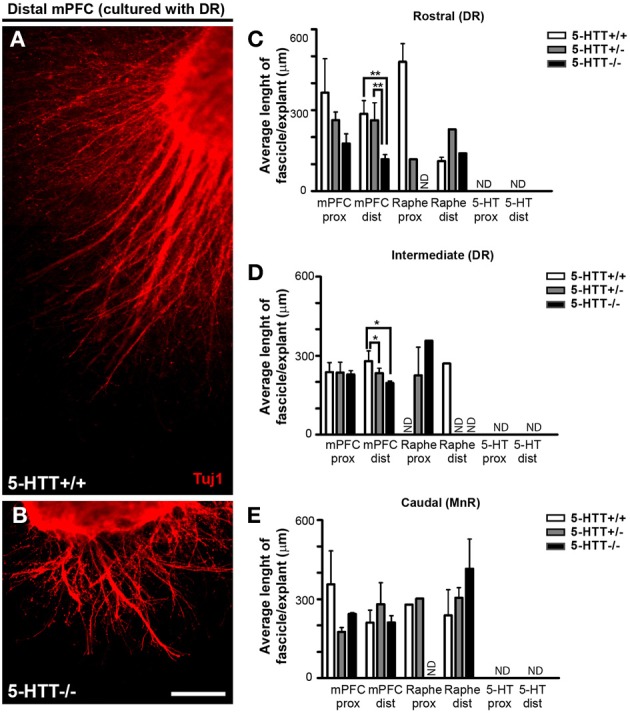
**Differences in length of fascicles from the mPFC controlled by the presence of 5-HTT during development. (A,B)** Example of differences in fascicle length in the distal quadrant of 5-HTT^+/+^ and 5-HTT^−/−^ mPFC co-cultured with the rostral subarea. Scale bar represents 100 μm. **(C–E)** Quantification of the length of fascicles in the proximal and distal quadrants of a subarea cocultured with mPFC. Graphs show average length of fascicles per explant ± SEM. One-Way ANOVA (α = 0.05), ^*^p < 0.05, ^**^p < 0.01.

To measure the length and width, we traced along and across the fascicle, resp. (Supplemental Figure 2B). We found differences in fascicle length growing from the mPFC when co-cultured with either the DR or the MnR, which depended on the genotype. Although not significant in the proximal quadrant of the mPFC, Figures 4A–D shows that in the distal quadrant of a wild-type or 5-HTT^+/−^ mPFC co-cultured with DR significantly longer fascicles were formed compared to the 5-HTT deficient situation (average length of fascicles, 286.3, 234.8 and 137.3 μm for HTT^+/+^, 5-HTT^+/−^, and 5-HTT^−/−^, resp.; *p* = 0.0097 and 0.0012, resp. for the rostral subarea and the average length of fascicles, 279.4, 221.9 and 195.5 μm for HTT^+/+^, 5-HTT^+/−^, and 5-HTT^−/−^, resp.; *p* = 0.049 and 0.012, resp. for the intermediate subarea). Small differences between DR and MnR were observed as well. For example, when comparing the distal quadrant of the rostral subarea of the DR (Figure 4C) with the MnR (Figure 4E) immunostained for β-III tubulin (all outgrowing neurites), the average fascicle length of fascicles from the MnR was longer.

Quantification of the average fascicle width among the various subareas and genotypes showed little significant differences, except for the proximal quadrant of the mPFC co-cultured with the rostral subarea of the DR where the width of the fascicles was significantly lower in the 5-HTT^−/−^ compared to fascicles of 5-HTT^+/−^ explants (average fascicle width is 12.03, 14.61, and 9.65 μm for HTT^+/+^, 5-HTT^+/−^, and 5-HTT^−/−^, resp.; *p* = 0.034; Supplemental Figure 3C). Supplemental Figures 3A,B illustrate the fascicle width of mPFC co-cultured with the MnR in 5-HTT^−/−^ compared to wild-type explants which was increased, although not significantly.

Taken together these data suggest that there are differences in the formation of fascicles by outgrowing neurites from the DR/MnR and from the mPFC, which seems to be affected by the (partial) lack of 5-HTT.

### Serotonergic innervation of various subareas of the mPFC is modulated by 5-HTT expression during development

**Figure 5:**
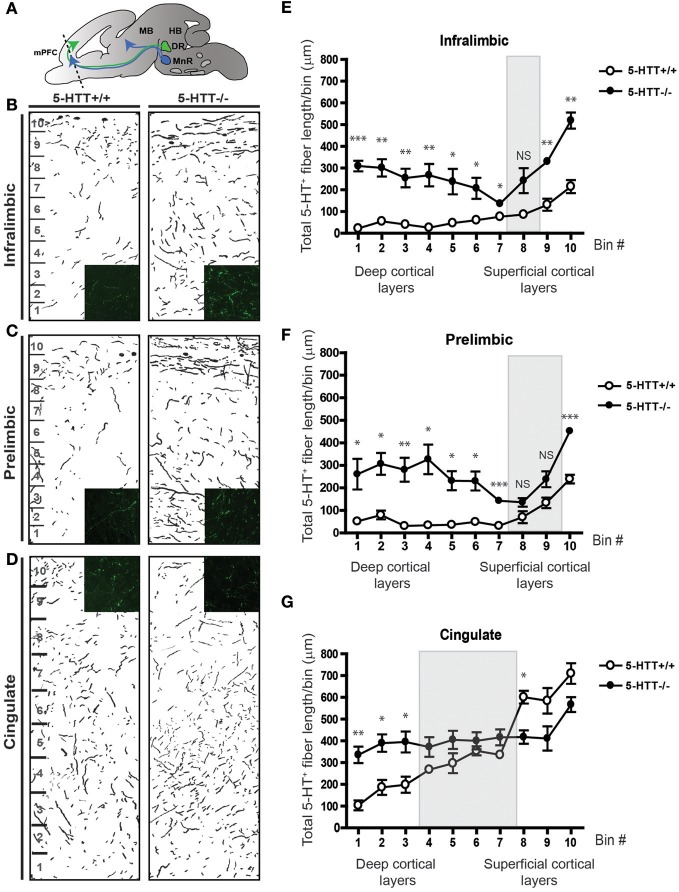
**Serotonergic innervation of the mPFC is increased in absence of the 5-HTT during development. (A)** Schematic showing the level of coronal sectioning (dotted line) within the mPFC comprised of the Infralimbic (IL, **B**), Prelimbic (PL, **C**) and Cingulate cortex (Cg, **D**). **(B)** Camera lucida drawings of 5-HT-positive fibers within the IL of 5-HTT^+/+^ and 5-HTT^−/−^ mPFC. Inset shows 5-HT fiber density (green) in the deeper layers of the mPFC. **(C)** Camera lucida drawings of 5-HT-positive fibers within the PL of 5-HTT^+/+^ and 5-HTT^−/−^ mPFC. Inset shows 5-HT fiber density (green) in the deeper layers of the mPFC. **(D)** Camera lucida drawings of 5-HT-positive fibers within the Cg of 5-HTT^+/+^ and 5-HTT^−/−^ mPFC. Inset shows 5-HT fiber density (green) in the superficial layers of the mPFC. **(E)** Quantification of the total 5-HT fiber length in μ m in 10 bins dividing the IL as indicated in **(B)** confirming the qualitative observations as seen in **(B)**. **(F)** Quantification of the total 5-HT fiber length in μ m in 10 bins dividing the PL as indicated in **(C)** confirming the qualitative observations as seen in **(C)**. **(G)** Quantification of the total 5-HT fiber length in μ m in 10 bins dividing the Cg as indicated in **(D)** confirming the qualitative observations as seen in **(D)**. There is a significant increase in the most deep cortical layers of the IL, PL as well as the Cg of 5-HTT^−/−^ compared to 5-HTT^+/+^ pups (*p* < 0.05–0.001). The gray boxes indicate the non-significant bins in the more superficial layers. Graphs show average length of 5-HT-positive fibers per bin ± SEM. One-Way ANOVA (α = 0.05), ^*^p < 0.05, ^**^p < 0.01, ^***^p < 0.001.

The ability of 5-HTT to regulate 5-HT levels during development (Buznikov et al., 2001; Narboux-Neme et al., 2008; Wiggins et al., 2012) together with the different directional responses of both raphe- as well as mPFC-derived projections raises the possibility that 5-HT and its signaling molecules can play an important role in axon guidance events navigating 5-HT axons to their forebrain targets. Therefore, we studied 5-HT innervation of the mPFC *in vivo* in 6 days old 5-HTT^−/−^ pups (P6) and compared that to wild-type innervation. Coronal sections of P6 5-HTT^−/−^ rats (*n* = 3) and wild-type littermate controls (*n* = 4) were stained for 5-HT to visualize raphe-derived projections and the length of the 5-HT innervation within the various subareas of the mPFC was measured. The length of the projections was quantified in 10 bins comprising the cerebral gray matter width (Figure 5). To better visualize 5-HT-positive fibers over the cerebral swatch containing the gray matter which included the deeper and most superficial cortical layers, we made camera lucida drawings (Figures 5B–D). In 5-HTT^−/−^ rats compared to control littermates, the drawings showed a clear increase in the amount of prefrontal 5-HT innervation in all subareas (Figures 5B–D). The average 5-HT-positive fiber length in the 5-HTT^−/−^ mPFC was higher as compared to the wild-type mPFC in every bin, except for bin 8 (IL) and bin 8 and 9 (PL), likely to represent layers II and III (Figures 5B,C,E,F). Remarkably, the cingulate mPFC showed a higher 5-HT fiber density in the deeper layers in absence of 5-HTT, whereas reduced levels were found in the more superficial layers (Figures 5D,G), suggesting another raphe-derived route of the developing 5-HT projections.

Overall, these results indicate a crucial developmental role for 5-HTT in the guidance of 5-HT projections to their targets in the mPFC.

### Absence of 5-HTT during development alters the identity of prefrontal projection neurons

**Figure 6:**
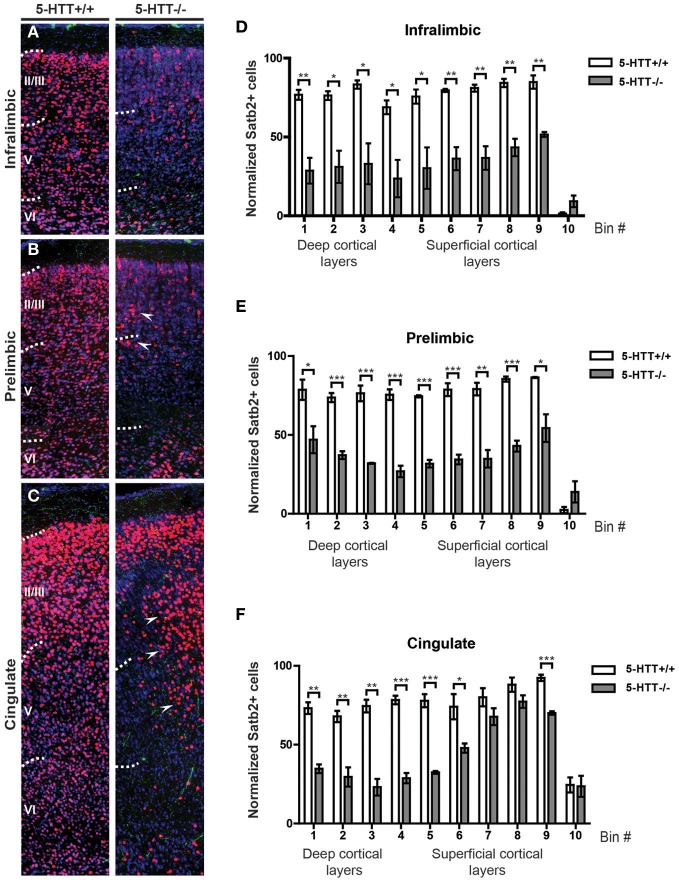
**Projection neuron identity is altered in absence of the 5-HTT during development. (A)** Coronal sections immunostained for 5-HT (green), Satb2 (red) and counterstained with fluorescent Nissl (blue) of 5-HTT^+/+^ and 5-HTT^−/−^ IL showing the position of callosal projection neurons (presumptive layer II-V). **(B)** Coronal sections of the PL as in **(A)**. **(C)** Coronal sections of the Cg as in **(A)**. Arrowheads indicate clusters of misplaced neurons. **(D)** Quantification of the number of Satb2-positive cells in 10 bins dividing the IL confirming the qualitative observations as seen in **(A)**. **(E)** Quantification of the number of Satb2-positive cells in 10 bins dividing the PL confirming the qualitative observations as seen in **(B)**. **(F)** Quantification of the number of Satb2-positive cells in 10 bins dividing the Cg confirming the qualitative observations as seen in **(C)**. There is a significant decrease in the number of Satb2-positive cells in the IL, PL, and Cg of 5-HTT^−/−^ compared to 5-HTT^+/+^ (*p* < 0.05–0.001). Graphs show average number of Satb2-positive cells per bin ± SEM. One-Way ANOVA (α = 0.05), ^*^p < 0.05, ^**^p < 0.01, ^***^p < 0.001.

Cortical neurons migrate to the proper location within the cortical plate (CP) through cell-autonomous and non-autonomous mechanisms (Kolk et al., 2009; Manent et al., 2011; Molnar and Clowry, 2012). To assess whether 5-HTT, either directly or indirectly, influences the identity and/or migration of prefrontal cortical neurons, we performed immunocytochemistry for both 5-HT and Satb2, a marker for upper-layer neurons (Dobreva et al., 2006; Alcamo et al., 2008; Britanova et al., 2008). Although the total number of neurons within the different subareas of the mPFC did not differ, we observed a remarkable reduction of Satb2-positive cells in all subareas of the mPFC in 5-HTT^−/−^ animals (*n* = 3) as compared to their wild-type littermates (*n* = 4) (Supplemental Figure 4). Surprisingly, Satb2-positive cells were no longer homogeneously restricted to layers II-VI, where a small percentage was scattered over the cerebral swatch in 5-HTT^−/−^ as compared to control mPFC (Figures 6A–F). Furthermore, it appeared that in the prelimbic but especially in the cingulate cortex of 5-HTT^−/−^ animals the Satb2-positive cells were often positioned in patches (Figures 6B,C, arrowheads). It remains to be established whether this reduction of Satb2-positive cells in the mPFC of rats lacking 5-HTT is cell-autonomous or due to increased 5-HT innervation, or both.

**Figure 7:**
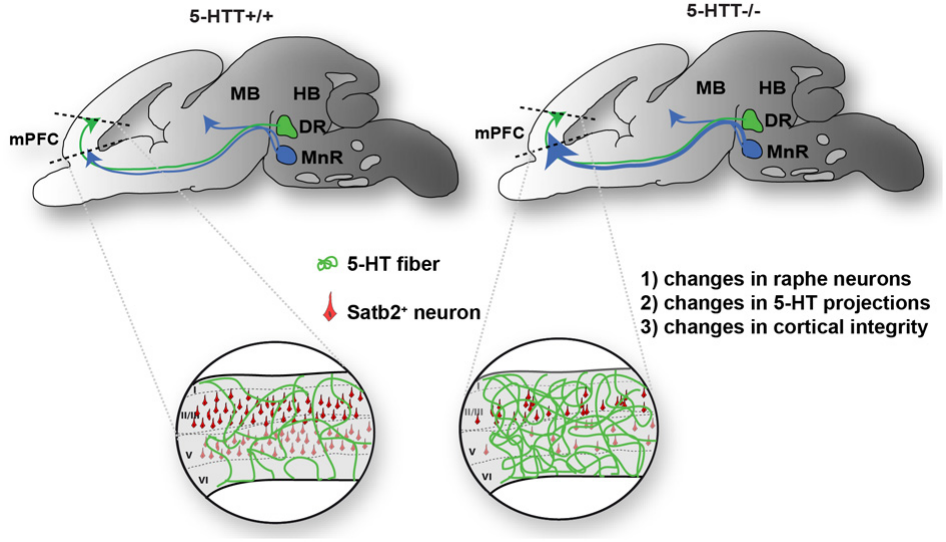
**Proposed model of the effect of 5-HTT deficiency on DR and MnR serotonergic neurite outgrowth during development.** Cg, cingulate cortex; DR, dorsal raphe nucleus; HB, hindbrain; IL, infralimbic; MB, midbrain; MnR, median raphe nucleus; PL, prelimbic.

In sum, these results indicate that appropriate 5-HTT levels during early brain development are important for proper maturation of the raphe-prefrontal projection system (Figure 7).

## Discussion

Our data show that the 5-HTT knockout rat represents an excellent model to investigate the role of 5-HTT in the development of the rostral raphe-prefrontal network formation. The present study evaluates the trophic nature of the interaction between the origin (rostral raphe cluster) and a target (mPFC) of the 5-HT projection system and how this interaction is modulated by the lack of the 5-HTT during development. Furthermore, we observed the ability of outgrowing neurites originating from the DR or MnR and mPFC to form fascicles, and once formed, we quantified the number, length, and width of the fascicles to evaluate the effect of 5-HTT expression during outgrowth. The nature of the interaction appears to depend on (1) the origin of 5-HT-positive projections within the rostral raphe cluster and (2) the presence of 5-HTT during development. In wild-type explants, the 5-HT fibers of the DR have a slightly attractive interaction with the mPFC, although not significant, while the 5-HT neurites of the MnR are repulsed by the mPFC. The most striking finding of this study shows that the 5-HT projections from the MnR become strongly attracted by the mPFC instead of being repelled in the absence of 5-HTT during development. *In vivo*, this is paralleled by the fact that in the 5-HTT^−/−^ mPFC the 5-HT innervation was significantly increased as compared to the wild-type situation. In addition we show that the number of Satb2-positive callosal projection neurons is reduced in absence of the 5-HTT. Together, these results lead us to hypothesize, as depicted in Figure 7, that due to lack of 5-HTT throughout development (1) the characteristics of the raphe neurons might have changed, (2) raphe neurons can have an altered guidance of their outgrowing neurites as well as (3) the identity of the neurons within the mPFC, which send out projections in their turn, may have changed.

### The role of 5-HTT in the development of the raphe nuclei

The 5-HTT is expressed in serotonergic neurons of the rostral raphe cluster as early as E11.5 (mouse) and E12.5 (rat) but also in non-serotonergic fibers such as thalamocortical projections (Schroeter and Blakely, 1996; Bengel et al., 1997; Bruning and Liangos, 1997; Bruning et al., 1997; Hansson et al., 1998; Zhou et al., 2000; Galineau et al., 2004; Narboux-Neme et al., 2008; Daws and Gould, 2011). One possibility to explain the observed results is that the characteristics of the 5-HT neurons within the rostral raphe have changed because of altered 5-HTT expression. Indeed, in absence of the 5-HTT, the DR neurons are fewer in number which could have an effect on the organization of the MnR and its projections (Lira et al., 2003). The 5-HT_1A_ of the DR neurons shows furthermore a marked desensitization when 5-HTT is lacking which could lead to functional consequences for the areas these DR axons innervate (Li et al., 2000; Mannoury La Cour et al., 2001; Holmes et al., 2003; Bose et al., 2011).

Three-dimensional collagen explant assays, are an excellent way to study interactions between areas within a particular network (Bonnin et al., 2007; Bozkurt et al., 2007; Kolk et al., 2009; Schmidt et al., 2012). When cultured alone, the various explants of the raphe and mPFC showed radial growth, indicating optimal growth conditions for the outgrowing neurites. Furthermore, we found specific and consistent outgrowth read-outs demonstrating the validity of the assay. Yet, there are several points of discussion when using sensitive *in vitro* assays like the explant assay. Although it was not measured in this particular study, we can assume that extracellular levels of 5-HT were elevated when the 5-HTT was absent (Gaspar et al., 2003; Riccio et al., 2009, 2011; Haenisch and Bonisch, 2011; Van Kleef et al., 2012). Even though the extrasynaptic concentration of 5-HT can reach the millimolar range (Bruns et al., 2000), the concentration and/or clearance of the released 5-HT within the medium was not measured over time in this experiment which could have had an effect on the observed results. In addition, the fact that a reduced 5-HT reuptake results in an increased 5-HT synthesis has been well documented (Kim et al., 2005; Haenisch and Bonisch, 2011). Although raphe explants from 5-HTT^−/−^/5-HTT^+/−^ animals showed 5-HT-positive outgrowing neurites, it is to be investigated how much 5-HT, or other soluble cues, is actually secreted by these neurons *in vitro*.

### Axonal guidance of serotonergic projections

The 5-HT projection system is one of the earliest neurotransmitter systems to innervate the brain, but the last to innervate the hippocampus and the cortex (Wilson and Molliver, 1991). During development, 5-HT may complete the maturation of a variety of neuronal projections systems, including its own, and the progression of interneuronal contacts (Lidov and Molliver, 1982; Daubert and Condron, 2010; Puig and Gulledge, 2011). The 5-HT neurons of the DR and MnR are known to innervate various subareas of the cortex including the mPFC (Bennett-Clarke et al., 1991; Del Cid-Pellitero and Garzon, 2011; Waselus et al., 2011; Celada et al., 2013; Chandler et al., 2013). While it cannot be excluded that the 5-HT raphe neurons are functionally different due to lack of 5-HTT, guidance of these and other (e.g., thalamocortical) projections might have been affected as well in absence of 5-HTT during development of the raphe-prefrontal network.

The observed interaction of the DR when co-cultured with the mPFC being neutral was unexpected since the DR is known to strongly innervate the cortical regions including the mPFC (Wilson and Molliver, 1991; Puig and Gulledge, 2011). Lack of 5-HTT did not affect the chemotropic nature of this interaction. This may indicate that another target of the 5-HT fibers lies beyond the mPFC and may encounter the mPFC as an intermediate target or needs an older mPFC to become attracted. Another possibility can be that the growing 5-HT projections need intermediate targets along its projection (e.g., thalamic regions) which is first encountered before projecting toward the cortical areas. An interesting question would be to evaluate whether the responses of the outgrowing neurites would change when E16.5 raphe tissue would be co-cultured with older mPFC explants or with other (intermediate) targets such as thalamus or hippocampus.

Interestingly, the interaction of the 5-HT fibers of the MnR toward the mPFC switched from a fairly strong repulsive to an attractive interaction in the absence of 5-HTT. *In vivo*, this could result in an increased innervation of the mPFC by the MnR. MnR-derived varicose M-fibers, which are believed to not express 5-HTT (Amilhon et al., 2010), mainly target layers II and III in the frontal parts of the cortex (Hensler et al., 2007), layers where we found the smallest differences in 5-HT innervation. In order to exclude the possibility that the switch of MnR neurites to attraction is due to an altered mPFC, it would be of interest to culture wild-type cortex together with DR/MnR from 5-HTT^+/−^ or 5-HTT^−/−^ animals.

Furthermore, 5-HT has been shown to have a modulatory effect on outgrowing axons by affecting their response to classical guidance cues such as netrin-1 (Bonnin et al., 2007). This means that in absence of the 5-HTT during development, the responses of the outgrowing neurites from either the raphe and/or the mPFC to guidance molecules may have altered. This would furthermore explain the *in vivo* results in that the 5-HT projections coming from the MnR are highly attracted by the mPFC when 5-HTT is lacking during development. It is plausible that because of the diminished expression of Satb2 (and maybe other transcription factors as well such as Ctip2), the expression of guidance cues is influenced. Indeed, it has been shown that the expression of a variety of axonal guidance molecules is amended in the absent expression of Satb2 (Alcamo et al., 2008). It would therefore be of interest to look for aberrant guidance cue expression in the 5-HTT^−/−^ animals and co-culture either the various subareas of the rostral raphe cluster, intermediate targets or the mPFC with HEK cells secreting an axonal guidance cue and to see whether the responsiveness of the outgrowing neurites would change in the absence of 5-HTT and/or could be modulated by 5-HT.

It has been shown that 5-HT plays a major role in the plasticity of the various other projection systems by modulating the guidance of their projections (Martin-Ruiz et al., 2001; Cunningham et al., 2005; Zhong et al., 2008). Since the overall outgrowth of neurites from the DR and MnR is affected by a (partial) lack of the 5-HTT, neurotransmitter systems other than the 5-HT system may be affected as well. Future experiments with 5-HTT^−/−^ three-dimensional explants of nuclei from other neurotransmitter systems such as the dopaminergic or noradrenergic system and their targets may give insight into such additional modulatory actions of 5-HT during development. While the total (Tuj1-positive) raphe and mPFC neurite outgrowth in the wild-type and 5-HTT^−/−^ situation appeared to be attractive by nature, this interaction becomes repulsive in the heterozygous animal. Perhaps, *in vitro*, either too high or too low concentrations of 5-HT permit an attractive response of both raphe as well as mPFC outgrowing neurites due to counteracting effects of different subtypes of 5-HT receptors expressed by the respective neurons (Celada et al., 2001; Martin-Ruiz et al., 2001; Ferezou et al., 2002; Santana et al., 2004; David et al., 2005; Alexandre et al., 2006; Riccio et al., 2009; Puig et al., 2010; Vucurovic et al., 2010), while intermediate 5-HT levels might elicit effects only via the more sensitive of the receptors producing a repulsive interaction. These results are intriguing considering that the s-allele carriers of the human 5-HTT polymorphism have a reduction in the amount of 5-HTT but never a complete lack (Lesch et al., 1996; Champoux et al., 2002; Pezawas et al., 2005; Homberg and Lesch, 2011).

### The function of 5-HTT in cortical integrity

A distant target of the ascending 5-HT projection system is the mPFC (D'Amato et al., 1987), which is involved in working memory and behavioral flexibility (Miller and Cohen, 2001) and is also one of the last brain areas to mature (Molnar and Clowry, 2012). The 5-HTT is the primary regulator of the 5-HT signal and may therefore affect the role of 5-HT in correct development of the brain. The presence of 5-HTT itself within the developing mPFC already starts from E14.5 onwards with 5-HTT-positive cells and later fibers innervating the mPFC in two parallel paths contacting many other cells (e.g., Cajal-Retzius cells) important for its correct development and layering (Lebrand et al., 1998; Zhou et al., 2000). Lack of 5-HTT during development could therefore have profound effects on the integrity of the mPFC itself.

The transcription factor Satb2 (special AT-rich sequence binding protein 2) is a marker exclusively expressed by callosal projection neurons from E13.5 onwards and Satb2-positive cells reside in cortical layers II-V and in subsets of neurons in layer VI (Dobreva et al., 2006; Alcamo et al., 2008; Britanova et al., 2008; Zhang et al., 2012). Satb2-positive callosal projection neurons were also present in the mPFC in which they most likely need selective 5-HT excitation to communicate (Avesar and Gulledge, 2012). In absence of the 5-HT transporter, the number of Satb2-positive neurons in the mPFC was decreased in all cortical layers, suggesting an altered identity of a set of prefrontal callosal projection neurons. Interestingly, there are indications that SSRIs given for the treatment of depression have an effect on the anatomy of the corpus callosum (Djavadian et al., 2003; Reyes-Haro et al., 2003; Simpson et al., 2011). In absence of Satb2, many downstream targets (e.g., guidance cues) are either up- or down-regulated in expression which can have an effect on the identity of a class of mPFC neurons themselves and/or have an effect on incoming projection systems (Alcamo et al., 2008). Since the 5-HT fibers are thought to contact the Cajal-Retzius (CR) cells in the marginal zone (presumptive cortical layer I) of the mPFC [(Janusonis et al., 2004; Chameau et al., 2009) and unpublished data], an increase in axonal innervation could result in an altered 5-HT signal onto the CR cells. The subsequent altered reelin release could have profound effects on the cortical layering resulting in a modified mPFC-mediated behavioral control. Therefore, we cannot rule out the possibility that during development, the migration of various classes of cortical projection neurons is also affected. To verify whether the callosal trajectory and/or identity of other projection neurons is affected in the absence of 5-HTT further experiments using various layer- and cell type-specific markers combined with electrophysiolological recordings are needed.

The excessive 5-HT fibers seen in the 5-HTT^−/−^ cortical plate can also have an effect on the maturity and dendritic arborization of the pyramidal neurons it harbors (Figure 6 and Miceli et al., 2013). Therefore it is likely that subsets of mPFC projections neurons have an altered identity due to altered expression levels of transcription factors (such as Satb2) in absence of 5-HTT during development. This can have consequences for later innervations of either serotonergic or other transmitter projections because of an altered permissive environment of the mPFC.

During the formation of long projections systems, scaffolds are formed by pioneer axons. The following axons can now trail these scaffolds toward their proper targets and associate together in fascicles (Van Vactor, 1998; Jaworski and Tessier-Lavigne, 2012). This is modulated by intrinsic membrane-bound cues and extrinsic diffusible cues. The number of mPFC explants of which neurites had formed fascicles was affected in the 5-HTT^−/−^ animals. This could indicate that the altered 5-HT signal influences the response of the mPFC neurites to the intrinsic membrane-bound and soluble cues influencing fasciculation. Additionally, the altered 5-HT signal could alter the actual cues, and thereby influence fasciculation (Petit et al., 2005). Altered fasciculation in the brain could have profound effects. For example in the case of defasciculation, fibers would be less densely packed and spread out over a larger area, with possible projections into surrounding areas that normally are not innervated. For future experiments, co-culturing mPFC of the different genotypes with wild-type raphe would shed some light on the role of an altered mPFC due to absence of 5-HTT in the development of the raphe-prefrontal network.

Because of the altered developmental 5-HT levels, one may wonder whether the expression of particular 5-HT receptors within the mPFC is altered (Li et al., 2003; Chameau et al., 2009; Riccio et al., 2009; Vucurovic et al., 2010), including 5-HT_1A_ and 5-HT_3A_ that are expressed by the CR cells themselves (Janusonis et al., 2004). The prefrontal pyramidal neurons express 5-HT_1A_, 5-HT_2A_ and 5-HT_6_ receptors (Celada et al., 2001; Martin-Ruiz et al., 2001; Santana et al., 2004; Riccio et al., 2009) and by changing the level of extracellular 5-HT, the conveyed signals differ and can result in an altered cell identity. The prefrontal neurons can show altered characteristics because of altered expression of 5-HTT in the mPFC itself (cell autonomous effect) or, due to the increased 5-HT innervation in the mPFC of 5-HTT^−/−^ animals (non-autonomous effect) this can lead to differences in cortical identity. For example, the amount of brain-derived-neurotrophic factor (BDNF) is dramatically decreased in the mPFC of animals lacking the 5-HTT (Mallo et al., 2008; Molteni et al., 2010), thereby perhaps changing the permissiveness of the mPFC environment to incoming projections. Therefore, experiments involving manipulations of 5-HT levels in the culture medium of the explants using receptor agonists (e.g., Flesinoxan or mCPBG), antagonists (eg WAY 100635 or tropisetron) or 5-HT itself, may provide additional information about the role of the 5-HT signal and the regulation hereof by the 5-HTT on the 5-HT neurite targeting and interaction with the mPFC (Ferezou et al., 2002; Alexandre et al., 2006; Chameau et al., 2009).

In sum, we conclude that the 5-HT projections arising from the rostral cluster of raphe and which innervate the mPFC seem to depend on the presence of 5-HTT during development. These results indicate that appropriate 5-HTT levels are required for proper 5-HT guidance, fasciculation, and innervation of the mPFC and that appropriate 5-HTT levels are important for proper development of the raphe-prefrontal projection system (Figure 7). Nevertheless, decoding the molecular program of 5-HT neurons and their projections toward the mPFC is a challenging task, and additional research is necessary. Given that 5-HTT^+/−^ animal models share many behavioral aspects with those seen in human 5-HTTLPR s-allele carriers, our data may help to understand the neurodevelopmental foundations of 5-HT associated behavioral phenotypes.

## Conflict of interest statement

The authors declare that the research was conducted in the absence of any commercial or financial relationships that could be construed as a potential conflict of interest.

## Supplementary material

Supplemental Figure 1 **Explant assays as a tool to study raphe-prefrontal network formation *in vitro*. (A)** Schematic representation showing the explant microdissection of the subareas of the rostral raphe cluster and the mPFC. The rostral raphe cluster was divided in a rostral (R), intermediate (I) and caudal (C) subarea and duplicated across the midline. Explants in a collagen hill were co-cultured at approximately 300 μm distance from each other. **(B)** Dorsal view of the dorsal raphe (DR) in a large explant showing 5-HT-positive neurons (green). **(B')** Enlargement of the boxed area in **(B)** showing the midline (dashed line) and individual 5-HT neurons sending out their projections. **(C)** Coronal cryosection showing 5-HT-positive neurons (green) in both the DR and MnR costained with Satb2 (red) and counterstained with fluorescent Nissl (blue). **(D)** DR explant stained for 5-HT (green) and Tuj1 (red) showing healthy axonal growth cones (asterisk and arrowheads).

Supplemental Figure 2 **Number of fascicles from the mPFC is moderated by the presence of 5-HTT during development. (A)** Fascicles coming from the mPFC and immunostained for Tuj1 (β-III tubulin) with a minimum width of 5 μm (arrows) the length and width were measured. Scale bar represents 50 μm. **(B)** Enlargement of the boxed area in **(A)**. To measure the length and width, we traced along and across the fascicle. **(C–E)** The percentage of explants with fascicles measured in the proximal quadrant per subarea and per genotype. **(F)** Examples of the proximal quadrant of the rostral subarea (DR) of the genotypes (5-HTT^+/+^, 5-HTT^+/−^and 5-HTT^−/−^) immunostained for 5-HT (green). No fascicles were formed by 5-HT-positive neurites growing from the DR.

Supplemental Figure 3 **Width of fascicles from the mPFC is modulated by the presence of 5-HTT during development. (A,B)** Examples of fascicles with a certain width in the distal quadrant of the MnR (caudal subarea) of wild-type and 5-HTT^−/−^ animals. Scale bar represents 20 μm. **(C–E)** Quantification of fascicle width for the different subareas and genotypes. Graphs show average length of fascicles per explant ± SEM. One-Way ANOVA (α = 0.05), ^*^*p* < 0.05.

Supplemental Figure 4 **Number of Satb2-positive neurons in the mPFC decrease in contrast to total number of cells in absence of 5-HTT during development. (A)** Schematic representation of the various subareas of the mPFC as quantified in **(B)**. **(B)** Quantification of the total number of cells (Nissl-positive) and the Satb2-positive cells in the various subareas of the mPFC over a swatch of 100 μm in width. Cg, cingulate cortex; IL, infralimbic; PL, prelimbic. Graphs show average number of cells per cortical swatch ± SEM. One-Way ANOVA (α = 0.05), ^*^*p* < 0.05, ^**^*p* < 0.01, ^***^*p* < 0.001.

## References

[B1] Abrams J. K. Johnson P. L. Hollis J. H. Lowry C. A. (2004). Anatomic and functional topography of the dorsal raphe nucleus. Ann. N.Y. Acad. Sci. 1018, 46–57 10.1196/annals.1296.005 15240351

[B2] Alcamo E. A. Chirivella L. Dautzenberg M. Dobreva G. Farinas I. Grosschedl R. (2008). Satb2 regulates callosal projection neuron identity in the developing cerebral cortex. Neuron 57, 364–377 10.1016/j.neuron.2007.12.012 18255030

[B3] Alexandre C. Popa D. Fabre V. Bouali S. Venault P. Lesch K. P. (2006). Early life blockade of 5-hydroxytryptamine 1A receptors normalizes sleep and depression-like behavior in adult knock-out mice lacking the serotonin transporter. J. Neurosci. 26, 5554–5564 10.1523/JNEUROSCI.5156-05.2006 16707806 PMC6675294

[B4] Alwan S. Friedman J. M. (2009). Safety of selective serotonin reuptake inhibitors in pregnancy. CNS Drugs 23, 493–509 10.2165/00023210-200923060-00004 19480468

[B5] Amilhon B. Lepicard E. Renoir T. Mongeau R. Popa D. Poirel O. (2010). VGLUT3 (vesicular glutamate transporter type 3) contribution to the regulation of serotonergic transmission and anxiety. J. Neurosci. 30, 2198–2210 10.1523/JNEUROSCI.5196-09.2010 20147547 PMC6634029

[B6] Anitha A. Nakamura K. Yamada K. Suda S. Thanseem I. Tsujii M. (2008). Genetic analyses of roundabout (ROBO) axon guidance receptors in autism. Am. J. Med. Genet. B Neuropsychiatr. Genet. 147B, 1019–1027 10.1002/ajmg.b.30697 18270976

[B7] Avesar D. Gulledge A. T. (2012). Selective serotonergic excitation of callosal projection neurons. Front. Neural Circuits 6:12 10.3389/fncir.2012.00012 22454619 PMC3308333

[B8] Balamotis M. A. Tamberg N. Woo Y. J. Li J. Davy B. Kohwi-Shigematsu T. (2012). Satb1 ablation alters temporal expression of immediate early genes and reduces dendritic spine density during postnatal brain development. Mol. Cell. Biol. 32, 333–347 10.1128/MCB.05917-11 22064485 PMC3255767

[B9] Bang S. J. Jensen P. Dymecki S. M. Commons K. G. (2012). Projections and interconnections of genetically defined serotonin neurons in mice. Eur. J. Neurosci. 35, 85–96 10.1111/j.1460-9568.2011.07936.x 22151329 PMC3268345

[B10] Barry J. Gu Y. Gu C. (2010). Polarized targeting of L1-CAM regulates axonal and dendritic bundling *in vitro.* Eur. J. Neurosci. 32, 1618–1631 10.1111/j.1460-9568.2010.07447.x 20964729 PMC2981701

[B11] Bengel D. Johren O. Andrews A. M. Heils A. Mossner R. Sanvitto G. L. (1997). Cellular localization and expression of the serotonin transporter in mouse brain. Brain Res. 778, 338–345 10.1016/S0006-8993(97)01080-9 9459551

[B12] Bennett-Clarke C. A. Chiaia N. L. Crissman R. S. Rhoades R. W. (1991). The source of the transient serotoninergic input to the developing visual and somatosensory cortices in rat. Neuroscience 43, 163–183 10.1016/0306-4522(91)90425-N 1656315

[B13] Bonnin A. Goeden N. Chen K. Wilson M. L. King J. Shih J. C. (2011). A transient placental source of serotonin for the fetal forebrain. Nature 472, 347–350 10.1038/nature09972 21512572 PMC3084180

[B14] Bonnin A. Levitt P. (2012). Placental source for 5-HT that tunes fetal brain development. Neuro-psychopharmacology 37, 299–300 10.1038/npp.2011.194 22157865 PMC3238076

[B15] Bonnin A. Torii M. Wang L. Rakic P. Levitt P. (2007). Serotonin modulates the response of embryonic thalamocortical axons to netrin-1. Nat. Neurosci. 10, 588–597 10.1038/nn1896 17450135

[B15a] Bose S. K. Mehta M. A. Selvaraj S. Howes O. D. Hinz R. Rabiner E. A. (2011). Presynaptic 5-HT1A is related to 5-HTT receptor density in the human brain. Neuropsychopharmacology 36, 2258–2265 10.1038/npp.2011.113 21750580 PMC3176562

[B16] Bozkurt A. Brook G. A. Moellers S. Lassner F. Sellhaus B. Weis J. (2007). *In vitro* assessment of axonal growth using dorsal root ganglia explants in a novel three-dimensional collagen matrix. Tissue Eng. 13, 2971–2979 10.1089/ten.2007.0116 17937537

[B17] Britanova O. De Juan Romero C. Cheung A. Kwan K. Y. Schwark M. Gyorgy A. (2008). Satb2 is a postmitotic determinant for upper-layer neuron specification in the neocortex. Neuron 57, 378–392 10.1016/j.neuron.2007.12.028 18255031

[B18] Bruning G. Liangos O. (1997). Transient expression of the serotonin transporter in the developing mouse thalamocortical system. Acta Histochem. 99, 117–121 10.1016/S0065-1281(97)80016-5 9150804

[B19] Bruning G. Liangos O. Baumgarten H. G. (1997). Prenatal development of the serotonin transporter in mouse brain. Cell Tissue Res. 289, 211–221 10.1007/s004410050868 9211824

[B20] Bruns D. Riedel D. Klingauf J. Jahn R. (2000). Quantal release of serotonin. Neuron 28, 205–220 10.1016/S0896-6273(00)00097-0 11086995

[B21] Buznikov G. A. Lambert H. W. Lauder J. M. (2001). Serotonin and serotonin-like substances as regulators of early embryogenesis and morphogenesis. Cell Tissue Res. 305, 177–186 10.1007/s004410100408 11545255

[B22] Canli T. Lesch K. P. (2007). Long story short: the serotonin transporter in emotion regulation and social cognition. Nat. Neurosci. 10, 1103–1109 10.1038/nn1964 17726476

[B23] Canli T. Omura K. Haas B. W. Fallgatter A. Constable R. T. Lesch K. P. (2005). Beyond affect: a role for genetic variation of the serotonin transporter in neural activation during a cognitive attention task. Proc. Natl. Acad. Sci. U.S.A. 102, 12224–12229 10.1073/pnas.0503880102 16093315 PMC1189322

[B24] Casper R. C. Gilles A. A. Fleisher B. E. Baran J. Enns G. Lazzeroni L. C. (2011). Length of prenatal exposure to selective serotonin reuptake inhibitor (SSRI) antidepressants: effects on neonatal adaptation and psychomotor development. Psychopharmacology (Berl.) 217, 211–219 10.1007/s00213-011-2270-z 21499702

[B25] Celada P. Puig M. V. Artigas F. (2013). Serotonin modulation of cortical neurons and networks. Front. Integr. Neurosci. 7:25 10.3389/fnint.2013.00025 23626526 PMC3630391

[B26] Celada P. Puig M. V. Casanovas J. M. Guillazo G. Artigas F. (2001). Control of dorsal raphe serotonergic neurons by the medial prefrontal cortex: involvement of serotonin-1A, GABA(A), and glutamate receptors. J. Neurosci. 21, 9917–9929 11739599 10.1523/JNEUROSCI.21-24-09917.2001 PMC6763042

[B27] Chameau P. Inta D. Vitalis T. Monyer H. Wadman W. J. Van Hooft J. A. (2009). The N-terminal region of reelin regulates postnatal dendritic maturation of cortical pyramidal neurons. Proc. Natl. Acad. Sci. U.S.A. 106, 7227–7232 10.1073/pnas.0810764106 19366679 PMC2678467

[B27a] Champoux M. Bennett A. Shannon C. Higley J. D. Lesch K. P. Suomi S. J. (2002). Serotonin transporter gene polymorphism, differential early rearing, and behavior in rhesus monkey neonates. Mol. Psychiatry 7, 1058–1063 10.1038/sj.mp.4001157 12476320

[B28] Chandler D. J. Lamperski C. S. Waterhouse B. D. (2013). Identification and distribution of projections from monoaminergic and cholinergic nuclei to functionally differentiated subregions of prefrontal cortex. Brain Res. 1522, 38–58 10.1016/j.brainres.2013.04.057 23665053 PMC3811940

[B29] Chugani D. C. Muzik O. Behen M. Rothermel R. Janisse J. J. Lee J. (1999). Developmental changes in brain serotonin synthesis capacity in autistic and nonautistic children. Ann. Neurol. 45, 287–295 10072042 10.1002/1531-8249(199903)45:3<287::aid-ana3>3.0.co;2-9

[B30] Costa L. Spatuzza M. D'antoni S. Bonaccorso C. M. Trovato C. Musumeci S. A. (2012). Activation of 5-HT7 serotonin receptors reverses metabotropic glutamate receptor-mediated synaptic plasticity in wild-type and Fmr1 knockout mice, a model of Fragile X syndrome. Biol. Psychiatry 72, 924–933 10.1016/j.biopsych.2012.06.008 22817866

[B31] Cote F. Fligny C. Bayard E. Launay J. M. Gershon M. D. Mallet J. (2007). Maternal serotonin is crucial for murine embryonic development. Proc. Natl. Acad. Sci. U.S.A. 104, 329–334 10.1073/pnas.0606722104 17182745 PMC1713169

[B32] Croen L. A. Grether J. K. Yoshida C. K. Odouli R. Hendrick V. (2011). Antidepressant use during pregnancy and childhood autism spectrum disorders. Arch. Gen. Psychiatry 68, 1104–1112 10.1001/archgenpsychiatry.2011.73 21727247

[B33] Cunningham M. G. Connor C. M. Zhang K. Benes F. M. (2005). Diminished serotonergic innervation of adult medial prefrontal cortex after 6-OHDA lesions in the newborn rat. Brain Res. Dev. Brain Res. 157, 124–131 10.1016/j.devbrainres.2005.02.020 15885807

[B34] Dahlstrom A. Fuxe K. (1964). Localization of monoamines in the lower brain stem. Experientia 20, 398–399 10.1007/BF02147990 5856530

[B35] D'Amato R. J. Blue M. E. Largent B. L. Lynch D. R. Ledbetter D. J. Molliver M. E. (1987). Ontogeny of the serotonergic projection to rat neocortex: transient expression of a dense innervation to primary sensory areas. Proc. Natl. Acad. Sci. U.S.A. 84, 4322–4326 10.1073/pnas.84.12.4322 3473503 PMC305077

[B36] Daubert E. A. Condron B. G. (2010). Serotonin: a regulator of neuronal morphology and circuitry. Trends Neurosci. 33, 424–434 10.1016/j.tins.2010.05.005 20561690 PMC2929308

[B37] David S. P. Murthy N. V. Rabiner E. A. Munafo M. R. Johnstone E. C. Jacob R. (2005). A functional genetic variation of the serotonin (5-HT) transporter affects 5-HT1A receptor binding in humans. J. Neurosci. 25, 2586–2590 10.1523/JNEUROSCI.3769-04.2005 15758168 PMC1942077

[B38] Daws L. C. Gould G. G. (2011). Ontogeny and regulation of the serotonin transporter: providing insights into human disorders. Pharmacol. Ther. 131, 61–79 10.1016/j.pharmthera.2011.03.013 21447358 PMC3131109

[B39] Del Cid-Pellitero E. Garzon M. (2011). Medial prefrontal cortex receives input from dorsal raphe nucleus neurons targeted by hypocretin1/orexinA-containing axons. Neuroscience 172, 30–43 10.1016/j.neuroscience.2010.10.058 21036204

[B40] Dickson B. J. (2002). Molecular mechanisms of axon guidance. Science 298, 1959–1964 10.1126/science.1072165 12471249

[B41] Djavadian R. L. Wielkopolska E. Turlejski K. (2003). Neonatal depletion of serotonin increases the numbers of callosally projecting neurons in cat visual areas 17 and 18. Neurosci. Lett. 351, 91–94 10.1016/j.neulet.2003.07.008 14583389

[B42] Dobreva G. Chahrour M. Dautzenberg M. Chirivella L. Kanzler B. Farinas I. (2006). SATB2 is a multifunctional determinant of craniofacial patterning and osteoblast differentiation. Cell 125, 971–986 10.1016/j.cell.2006.05.012 16751105

[B43] Fenstermaker A. G. Prasad A. A. Bechara A. Adolfs Y. Tissir F. Goffinet A. (2010). Wnt/planar cell polarity signaling controls the anterior-posterior organization of monoaminergic axons in the brainstem. J. Neurosci. 30, 16053–16064 10.1523/JNEUROSCI.4508-10.2010 21106844 PMC3073573

[B44] Ferezou I. Cauli B. Hill E. L. Rossier J. Hamel E. Lambolez B. (2002). 5-HT3 receptors mediate serotonergic fast synaptic excitation of neocortical vasoactive intestinal peptide/cholecystokinin interneurons. J. Neurosci. 22, 7389–7397 12196560 10.1523/JNEUROSCI.22-17-07389.2002 PMC6757992

[B45] Galineau L. Kodas E. Guilloteau D. Vilar M. P. Chalon S. (2004). Ontogeny of the dopamine and serotonin transporters in the rat brain: an autoradiographic study. Neurosci. Lett. 363, 266–271 10.1016/j.neulet.2004.04.007 15182957

[B46] Gaspar P. Cases O. Maroteaux L. (2003). The developmental role of serotonin: news from mouse molecular genetics. Nat. Rev. Neurosci. 4, 1002–1012 10.1038/nrn1256 14618156

[B47] Gentile S. Galbally M. (2011). Prenatal exposure to antidepressant medications and neurodevelopmental outcomes: a systematic review. J. Affect. Disord. 128, 1–9 10.1016/j.jad.2010.02.125 20303599

[B48] Gurevich E. V. Joyce J. N. (1997). Alterations in the cortical serotonergic system in schizophrenia: a postmortem study. Biol. Psychiatry 42, 529–545 10.1016/S0006-3223(97)00321-1 9376449

[B49] Haddley K. Bubb V. J. Breen G. Parades-Esquivel U. M. Quinn J. P. (2012). Behavioural genetics of the serotonin transporter. Curr. Top. Behav. Neurosci. . [Epub ahead of print]. 10.1007/7854_2011_186 22261701

[B50] Haenisch B. Bonisch H. (2011). Depression and antidepressants: insights from knockout of dopamine, serotonin or noradrenaline re-uptake transporters. Pharmacol. Ther. 129, 352–368 10.1016/j.pharmthera.2010.12.002 21147164

[B51] Hansson S. R. Mezey E. Hoffman B. J. (1998). Serotonin transporter messenger RNA in the developing rat brain: early expression in serotonergic neurons and transient expression in non-serotonergic neurons. Neuroscience 83, 1185–1201 10.1016/S0306-4522(97)00444-2 9502257

[B52] Heidbreder C. A. Groenewegen H. J. (2003). The medial prefrontal cortex in the rat: evidence for a dorso-ventral distinction based upon functional and anatomical characteristics. Neurosci. Biobehav. Rev. 27, 555–579 10.1016/j.neubiorev.2003.09.003 14599436

[B53] Hensler J. G. Advani T. Monteggia L. M. (2007). Regulation of serotonin-1A receptor function in inducible brain-derived neurotrophic factor knockout mice after administration of corticosterone. Biol. Psychiatry 62, 521–529 10.1016/j.biopsych.2006.10.015 17336942

[B53a] Holmes A. Yang R. J. Lesch K. P. Crawley J. N. Murphy D. L. (2003). Mice lacking the serotonin transporter exhibit 5-HT(1A) receptor-mediated abnormalities in tests for anxiety-like behavior. Neuropsychopharmacology 28, 2077–2088 10.1038/sj.npp.1300266 12968128

[B53b] Homberg J. R. Lesch K. P. (2011). Looking on the bright side of serotonin transporter gene variation. Biol. Psychiatry 69, 513–519 10.1016/j.biopsych.2010.09.024 21047622

[B54] Homberg J. R. Olivier J. D. Smits B. M. Mul J. D. Mudde J. Verheul M. (2007a). Characterization of the serotonin transporter knockout rat: a selective change in the functioning of the serotonergic system. Neuroscience 146, 1662–1676 17467186 10.1016/j.neuroscience.2007.03.030

[B55] Homberg J. R. Pattij T. Janssen M. C. Ronken E. De Boer S. F. Schoffelmeer A. N. (2007b). Serotonin transporter deficiency in rats improves inhibitory control but not behavioural flexibility. Eur. J. Neurosci. 26, 2066–2073 17897403 10.1111/j.1460-9568.2007.05839.x

[B56] Homberg J. R. Schubert D. Gaspar P. (2010). New perspectives on the neurodevelopmental effects of SSRIs. Trends Pharmacol. Sci. 31, 60–65 10.1016/j.tips.2009.11.003 19963284

[B57] Janusonis S. Gluncic V. Rakic P. (2004). Early serotonergic projections to Cajal-Retzius cells: relevance for cortical development. J. Neurosci. 24, 1652–1659 10.1523/JNEUROSCI.4651-03.2004 14973240 PMC6730467

[B58] Jaworski A. Tessier-Lavigne M. (2012). Autocrine/juxtaparacrine regulation of axon fasciculation by Slit-Robo signaling. Nat. Neurosci. 15, 367–369 10.1038/nn.3037 22306607

[B59] Kalueff A. V. Olivier J. D. Nonkes L. J. Homberg J. R. (2010). Conserved role for the serotonin transporter gene in rat and mouse neurobehavioral endophenotypes. Neurosci. Biobehav. Rev. 34, 373–386 10.1016/j.neubiorev.2009.08.003 19698744

[B60] Kim D. K. Tolliver T. J. Huang S. J. Martin B. J. Andrews A. M. Wichems C. (2005). Altered serotonin synthesis, turnover and dynamic regulation in multiple brain regions of mice lacking the serotonin transporter. Neuropharmacology 49, 798–810 10.1016/j.neuropharm.2005.08.010 16183083

[B61] Kolk S. M. Gunput R. A. Tran T. S. Van Den Heuvel D. M. Prasad A. A. Hellemons A. J. (2009). Semaphorin 3F is a bifunctional guidance cue for dopaminergic axons and controls their fasciculation, channeling, rostral growth, and intracortical targeting. J. Neurosci. 29, 12542–12557 10.1523/JNEUROSCI.2521-09.2009 19812329 PMC3097132

[B62] Kolk S. M. Whitman M. C. Yun M. E. Shete P. Donoghue M. J. (2006). A unique subpopulation of Tbr1-expressing deep layer neurons in the developing cerebral cortex. Mol. Cell. Neurosci. 32, 200–214 10.1016/j.mcn.2005.08.022 16858776

[B63] Lauder J. M. (1990). Ontogeny of the serotonergic system in the rat: serotonin as a developmental signal. Ann. N.Y. Acad. Sci. 600, 297–313 discussion: 314. 2252317 10.1111/j.1749-6632.1990.tb16891.x

[B64] Lebrand C. Cases O. Wehrle R. Blakely R. D. Edwards R. H. Gaspar P. (1998). Transient developmental expression of monoamine transporters in the rodent forebrain. J. Comp. Neurol. 401, 506–524 9826275

[B65] Lee J. K. Chow R. Xie F. Chow S. Y. Tolentino K. E. Zheng B. (2010). Combined genetic attenuation of myelin and semaphorin-mediated growth inhibition is insufficient to promote serotonergic axon regeneration. J. Neurosci. 30, 10899–10904 10.1523/JNEUROSCI.2269-10.2010 20702718 PMC2974627

[B66] Leemhuis J. Bouche E. Frotscher M. Henle F. Hein L. Herz J. (2010). Reelin signals through apolipoprotein E receptor 2 and Cdc42 to increase growth cone motility and filopodia formation. J. Neurosci. 30, 14759–14772 10.1523/JNEUROSCI.4036-10.2010 21048135 PMC3523719

[B66b] Lesch K. P. Bengel D. Heils A. Sabol S. Z. Greenberg B. D. Petri S. (1996). Association of anxiety-related traits with a polymorphism in the serotonin transporter gene regulatory region. Science 274, 1527–1531 10.1126/science.274.5292.1527 8929413

[B66a] Li Q. Wichems C. Heils A. Lesch K. P. Murphy D. L. (2000). Reduction in the density and expression, but not G-protein coupling, of serotonin receptors (5-HT_1A_) in 5-HT transporter knock-out mice: gender and brain region differences. J. Neurosci. 20, 7888–7895 11050108 10.1523/JNEUROSCI.20-21-07888.2000 PMC6772750

[B67] Li Q. Wichems C. H. Ma L. Van De Kar L. D. Garcia F. Murphy D. L. (2003). Brain region-specific alterations of 5-HT2A and 5-HT2C receptors in serotonin transporter knockout mice. J. Neurochem. 84, 1256–1265 10.1046/j.1471-4159.2003.01607.x 12614326

[B68] Lidov H. G. Molliver M. E. (1982). An immunohistochemical study of serotonin neuron development in the rat: ascending pathways and terminal fields. Brain Res. Bull. 8, 389–430 10.1016/0361-9230(82)90077-6 6178481

[B69] Lira A. Zhou M. Castanon N. Ansorge M. S. Gordon J. A. Francis J. H. (2003). Altered depression-related behaviors and functional changes in the dorsal raphe nucleus of serotonin transporter-deficient mice. Biol. Psychiatry 54, 960–971 10.1016/S0006-3223(03)00696-6 14625138

[B70] Mallo T. Koiv K. Koppel I. Raudkivi K. Uustare A. Rinken A. (2008). Regulation of extracellular serotonin levels and brain-derived neurotrophic factor in rats with high and low exploratory activity. Brain Res. 1194, 110–117 10.1016/j.brainres.2007.11.041 18177844 PMC2568862

[B71] Manent J. B. Beguin S. Ganay T. Represa A. (2011). Cell-autonomous and cell-to-cell signalling events in normal and altered neuronal migration. Eur. J. Neurosci. 34, 1595–1608 10.1111/j.1460-9568.2011.07867.x 22103417

[B72] Mann J. J. (2013). The serotonergic system in mood disorders and suicidal behaviour. Philos. Trans. R. Soc. Lond. B. Biol. Sci. 368:20120537 10.1098/rstb.2012.0537 23440471 PMC3638390

[B72a] Mannoury La Cour C. Boni C. Hanoun N. Lesch K. P. Hamon M. Lanfumey L. (2001). Functional consequences of 5-HT transporter gene disruption on 5-HT_(1a_) receptor-mediated regulation of dorsal raphe and hippocampal cell activity. J. Neurosci. 21, 2178–2185 11245702 10.1523/JNEUROSCI.21-06-02178.2001 PMC6762595

[B73] Martin-Ruiz R. Puig M. V. Celada P. Shapiro D. A. Roth B. L. Mengod G. (2001). Control of serotonergic function in medial prefrontal cortex by serotonin-2A receptors through a glutamate-dependent mechanism. J. Neurosci. 21, 9856–9866 11739593 10.1523/JNEUROSCI.21-24-09856.2001 PMC6763049

[B73a] Miceli S. Negwer M. Van Eijs F. Kalkhoven C. Van Lierop I. Homberg J. (2013). High serotonin levels during brain development alter the structural input-output connectivity of neural networks in the rat somatosensory layer IV. Front. Cell Neurosci. 7:88 10.3389/fncel.2013.00088 23761736 PMC3675331

[B74] Migliarini S. Pacini G. Pelosi B. Lunardi G. Pasqualetti M. (2012). Lack of brain serotonin affects postnatal development and serotonergic neuronal circuitry formation. Mol. Psychiatry. . [Epub ahead of print]. 10.1038/mp.2012.128 23007167

[B75] Miller E. K. Cohen J. D. (2001). An integrative theory of prefrontal cortex function. Annu. Rev. Neurosci. 24, 167–202 10.1146/annurev.neuro.24.1.167 11283309

[B76] Molnar Z. Clowry G. (2012). Cerebral cortical development in rodents and primates. Prog. Brain Res. 195, 45–70 10.1016/B978-0-444-53860-4.00003-9 22230622

[B77] Molteni R. Cattaneo A. Calabrese F. Macchi F. Olivier J. D. Racagni G. (2010). Reduced function of the serotonin transporter is associated with decreased expression of BDNF in rodents as well as in humans. Neurobiol. Dis. 37, 747–755 10.1016/j.nbd.2009.12.014 20034565

[B78] Narboux-Neme N. Pavone L. M. Avallone L. Zhuang X. Gaspar P. (2008). Serotonin transporter transgenic (SERTcre) mouse line reveals developmental targets of serotonin specific reuptake inhibitors (SSRIs). Neuropharmacology 55, 994–1005 10.1016/j.neuropharm.2008.08.020 18789954

[B79] Neumann I. D. Wegener G. Homberg J. R. Cohen H. Slattery D. A. Zohar J. (2011). Animal models of depression and anxiety: what do they tell us about human condition? Prog. Neuropsychopharmacol. Biol. Psychiatry 35, 1357–1375 21129431 10.1016/j.pnpbp.2010.11.028

[B80] Oberlander T. F. Gingrich J. A. Ansorge M. S. (2009). Sustained neurobehavioral effects of exposure to SSRI antidepressants during development: molecular to clinical evidence. Clin. Pharmacol. Ther. 86, 672–677 10.1038/clpt.2009.201 19890255 PMC3963518

[B81] Pasterkamp R. J. Peschon J. J. Spriggs M. K. Kolodkin A. L. (2003). Semaphorin 7A promotes axon outgrowth through integrins and MAPKs. Nature 424, 398–405 10.1038/nature01790 12879062

[B82] Petit A. Kennedy T. E. Bagnard D. Doucet G. (2005). Membrane-associated guidance cues direct the innervation of forebrain and midbrain by dorsal raphe-derived serotonergic axons. Eur. J. Neurosci. 22, 552–568 10.1111/j.1460-9568.2005.04249.x 16101737

[B82a] Pezawas L. Meyer-Lindenberg A. Drabant E. M. Verchinski B. A. Munoz K. E. Kolachana B. S. (2005). 5-HTTLPR polymorphism impacts human cingulate-amygdala interactions: a genetic susceptibility mechanism for depression. Nat. Neurosci. 8, 828–834 10.1038/nn1463 15880108

[B83] Puig M. V. Gulledge A. T. (2011). Serotonin and prefrontal cortex function: neurons, networks, and circuits. Mol. Neurobiol. 44, 449–464 10.1007/s12035-011-8214-0 22076606 PMC3282112

[B84] Puig M. V. Watakabe A. Ushimaru M. Yamamori T. Kawaguchi Y. (2010). Serotonin modulates fast-spiking interneuron and synchronous activity in the rat prefrontal cortex through 5-HT1A and 5-HT2A receptors. J. Neurosci. 30, 2211–2222 10.1523/JNEUROSCI.3335-09.2010 20147548 PMC6634052

[B85] Rampono J. Proud S. Hackett L. P. Kristensen J. H. Ilett K. F. (2004). A pilot study of newer antidepressant concentrations in cord and maternal serum and possible effects in the neonate. Int. J. Neuropsychopharmacol. 7, 329–334 10.1017/S1461145704004286 15035694

[B86] Reyes-Haro D. Garcia-Alcocer G. Miledi R. Garcia-Colunga J. (2003). Uptake of serotonin by adult rat corpus callosum is partially reduced by common antidepressants. J. Neurosci. Res. 74, 97–102 10.1002/jnr.10724 13130511

[B87] Riccio O. Jacobshagen M. Golding B. Vutskits L. Jabaudon D. Hornung J. P. (2011). Excess of serotonin affects neocortical pyramidal neuron migration. Transl. Psychiatry 1:e47 10.1038/tp.2011.49 22833193 PMC3309486

[B88] Riccio O. Potter G. Walzer C. Vallet P. Szabo G. Vutskits L. (2009). Excess of serotonin affects embryonic interneuron migration through activation of the serotonin receptor 6. Mol. Psychiatry 14, 280–290 10.1038/mp.2008.89 18663366

[B89] Robbins T. W. Arnsten A. F. (2009). The neuropsychopharmacology of fronto-executive function: monoaminergic modulation. Annu. Rev. Neurosci. 32, 267–287 10.1146/annurev.neuro.051508.135535 19555290 PMC2863127

[B90] Santana N. Bortolozzi A. Serrats J. Mengod G. Artigas F. (2004). Expression of serotonin1A and serotonin2A receptors in pyramidal and GABAergic neurons of the rat prefrontal cortex. Cereb. Cortex 14, 1100–1109 10.1093/cercor/bhh070 15115744

[B91] Schmidt E. R. Morello F. Pasterkamp R. J. (2012). Dissection and culture of mouse dopaminergic and striatal explants in three-dimensional collagen matrix assays. J. Vis. Exp. pii:3691 10.3791/3691 22473326 PMC3460578

[B92] Schroeter S. Blakely R. D. (1996). Drug targets in the embryo. Studies on the cocaine- and antidepressant-sensitive serotonin transporter. Ann. N.Y. Acad. Sci. 801, 239–255 10.1111/j.1749-6632.1996.tb17446.x 8959038

[B93] Simpson K. L. Weaver K. J. De Villers-Sidani E. Lu J. Y. Cai Z. Pang Y. (2011). Perinatal antidepressant exposure alters cortical network function in rodents. Proc. Natl. Acad. Sci. U.S.A. 108, 18465–18470 10.1073/pnas.1109353108 22025710 PMC3215047

[B94] Smits B. M. Mudde J. B. Van De Belt J. Verheul M. Olivier J. Homberg J. (2006). Generation of gene knockouts and mutant models in the laboratory rat by ENU-driven target-selected mutagenesis. Pharmacogenet. Genomics 16, 159–169 16495775 10.1097/01.fpc.0000184960.82903.8f

[B95] Sodhi M. S. Sanders-Bush E. (2004). Serotonin and brain development. Int. Rev. Neurobiol. 59, 111–174 10.1016/S0074-7742(04)59006-2 15006487

[B96] Souza B. R. Tropepe V. (2011). The role of dopaminergic signalling during larval zebrafish brain development: a tool for investigating the developmental basis of neuropsychiatric disorders. Rev. Neurosci. 22, 107–119 21615265 10.1515/RNS.2011.012

[B97] Tessier-Lavigne M. Goodman C. S. (1996). The molecular biology of axon guidance. Science 274, 1123–1133 10.1126/science.274.5290.1123 8895455

[B98] Van Bockstaele E. J. Biswas A. Pickel V. M. (1993). Topography of serotonin neurons in the dorsal raphe nucleus that send axon collaterals to the rat prefrontal cortex and nucleus accumbens. Brain Res. 624, 188–198 10.1016/0006-8993(93)90077-Z 8252391

[B99] Van Kleef E. S. Gaspar P. Bonnin A. (2012). Insights into the complex influence of 5-HT signaling on thalamocortical axonal system development. Eur. J. Neurosci. 35, 1563–1572 10.1111/j.1460-9568.2012.8096.x 22607002 PMC3359868

[B100] Van Vactor D. (1998). Adhesion and signaling in axonal fasciculation. Curr. Opin. Neurobiol. 8, 80–86 10.1016/S0959-4388(98)80011-1 9568395

[B101] Verney C. Lebrand C. Gaspar P. (2002). Changing distribution of monoaminergic markers in the developing human cerebral cortex with special emphasis on the serotonin transporter. Anat. Rec. 267, 87–93 10.1002/ar.10089 11997877

[B102] Vitalis T. Cases O. Passemard S. Callebert J. Parnavelas J. G. (2007). Embryonic depletion of serotonin affects cortical development. Eur. J. Neurosci. 26, 331–344 10.1111/j.1460-9568.2007.05661.x 17650110

[B103] Vucurovic K. Gallopin T. Ferezou I. Rancillac A. Chameau P. Van Hooft J. A. (2010). Serotonin 3A receptor subtype as an early and protracted marker of cortical interneuron subpopulations. Cereb. Cortex 20, 2333–2347 10.1093/cercor/bhp310 20083553 PMC2936799

[B104] Waselus M. Valentino R. J. Van Bockstaele E. J. (2011). Collateralized dorsal raphe nucleus projections: a mechanism for the integration of diverse functions during stress. J. Chem. Neuroanat. 41, 266–280 10.1016/j.jchemneu.2011.05.011 21658442 PMC3156417

[B105] Whitaker-Azmitia P. M. (2001). Serotonin and brain development: role in human developmental diseases. Brain Res. Bull. 56, 479–485 10.1016/S0361-9230(01)00615-3 11750793

[B106] Whitaker-Azmitia P. M. (2005). Behavioral and cellular consequences of increasing serotonergic activity during brain development: a role in autism? Int. J. Dev. Neurosci. 23, 75–83 10.1016/j.ijdevneu.2004.07.022 15730889

[B107] Whitaker-Azmitia P. M. Druse M. Walker P. Lauder J. M. (1996). Serotonin as a developmental signal. Behav. Brain Res. 73, 19–29 10.1016/0166-4328(96)00071-X 8788472

[B108] Wiggins J. L. Bedoyan J. K. Peltier S. J. Ashinoff S. Carrasco M. Weng S. J. (2012). The impact of serotonin transporter (5-HTTLPR) genotype on the development of resting-state functional connectivity in children and adolescents: a preliminary report. Neuroimage 59, 2760–2770 10.1016/j.neuroimage.2011.10.030 22032950 PMC3254835

[B109] Wilson M. A. Molliver M. E. (1991). The organization of serotonergic projections to cerebral cortex in primates: retrograde transport studies. Neuroscience 44, 555–570 10.1016/0306-4522(91)90077-2 1721683

[B110] Zhang L. Song N. N. Chen J. Y. Huang Y. Li H. Ding Y. Q. (2012). Satb2 is required for dendritic arborization and soma spacing in mouse cerebral cortex. Cereb. Cortex 22, 1510–1519 10.1093/cercor/bhr215 21885532

[B111] Zhong P. Liu W. Gu Z. Yan Z. (2008). Serotonin facilitates long-term depression induction in prefrontal cortex via p38 MAPK/Rab5-mediated enhancement of AMPA receptor internalization. J. Physiol. (Lond.) 586, 4465–4479 10.1113/jphysiol.2008.155143 18653660 PMC2614015

[B112] Zhou F. C. Sari Y. Zhang J. K. (2000). Expression of serotonin transporter protein in developing rat brain. Brain Res. Dev. Brain Res. 119, 33–45 10.1016/S0165-3806(99)00152-2 10648870

