# Supplementary material for: Lack of serotonin reuptake during brain development alters rostral raphe-prefrontal network formation
Source: Front Cell Neurosci. 2013 Oct 4;7:143. doi: 10.3389/fncel.2013.00143 (PMC3790074; doi:10.3389/fncel.2013.00143)

Witteveen *et al.*, Supplemental Figure 1

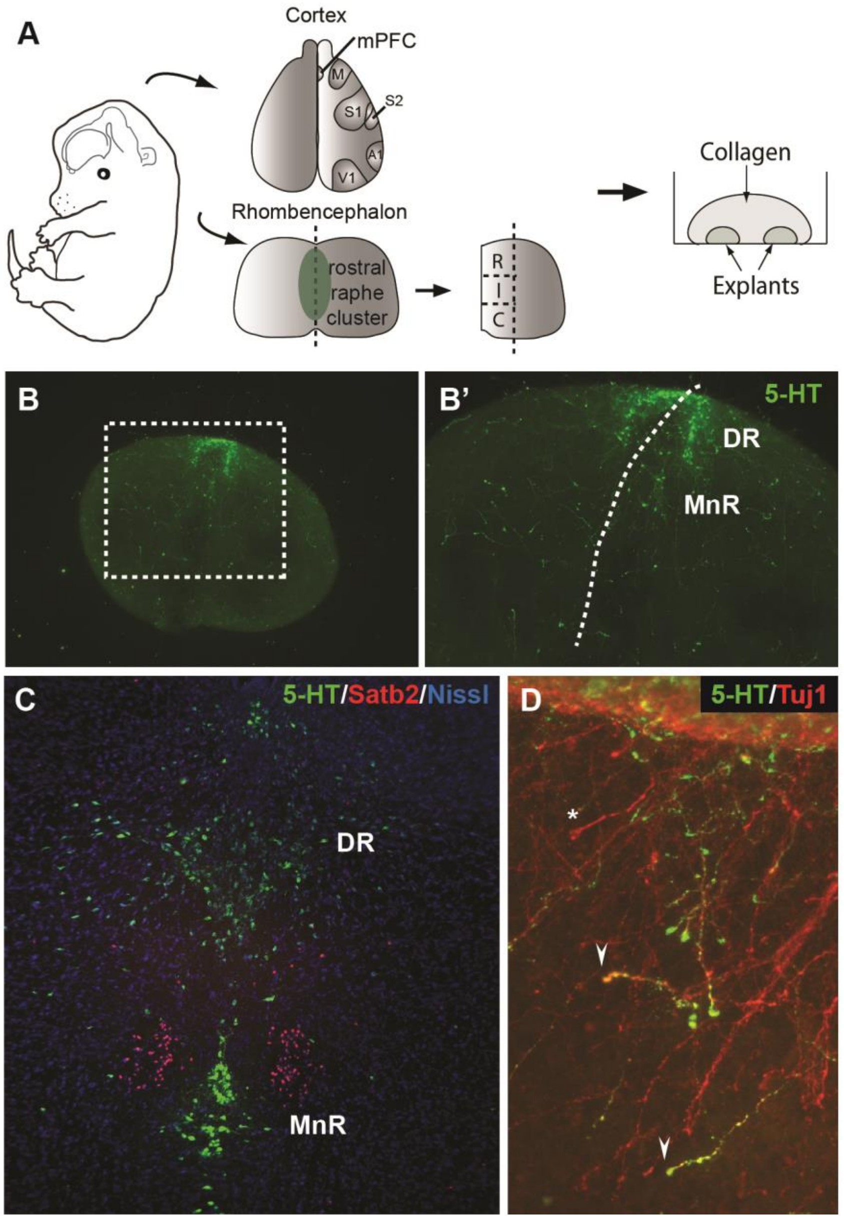

Witteveen et al., Supplemental Figure 2

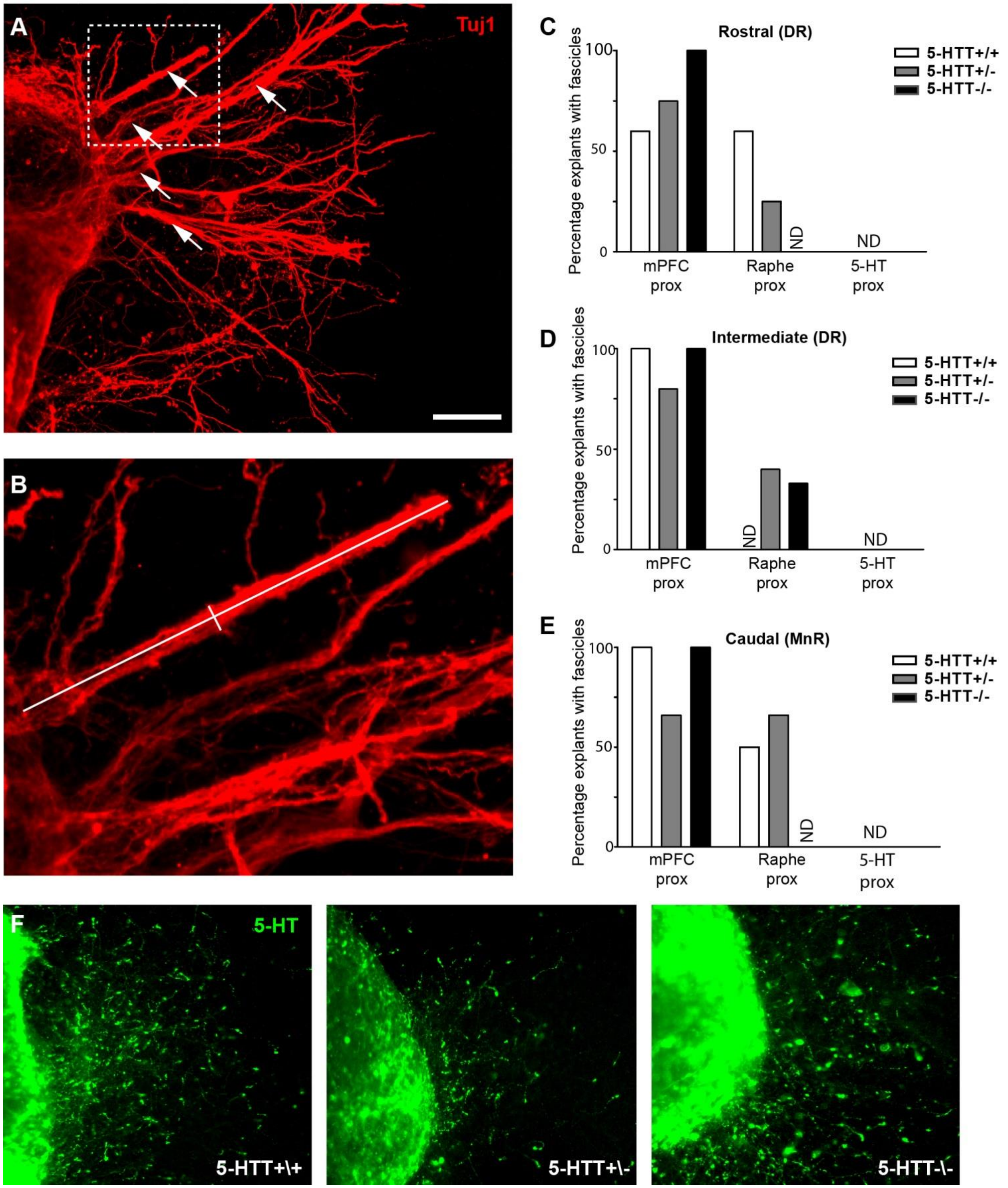

Witteveen et al., Supplemental Figure 3

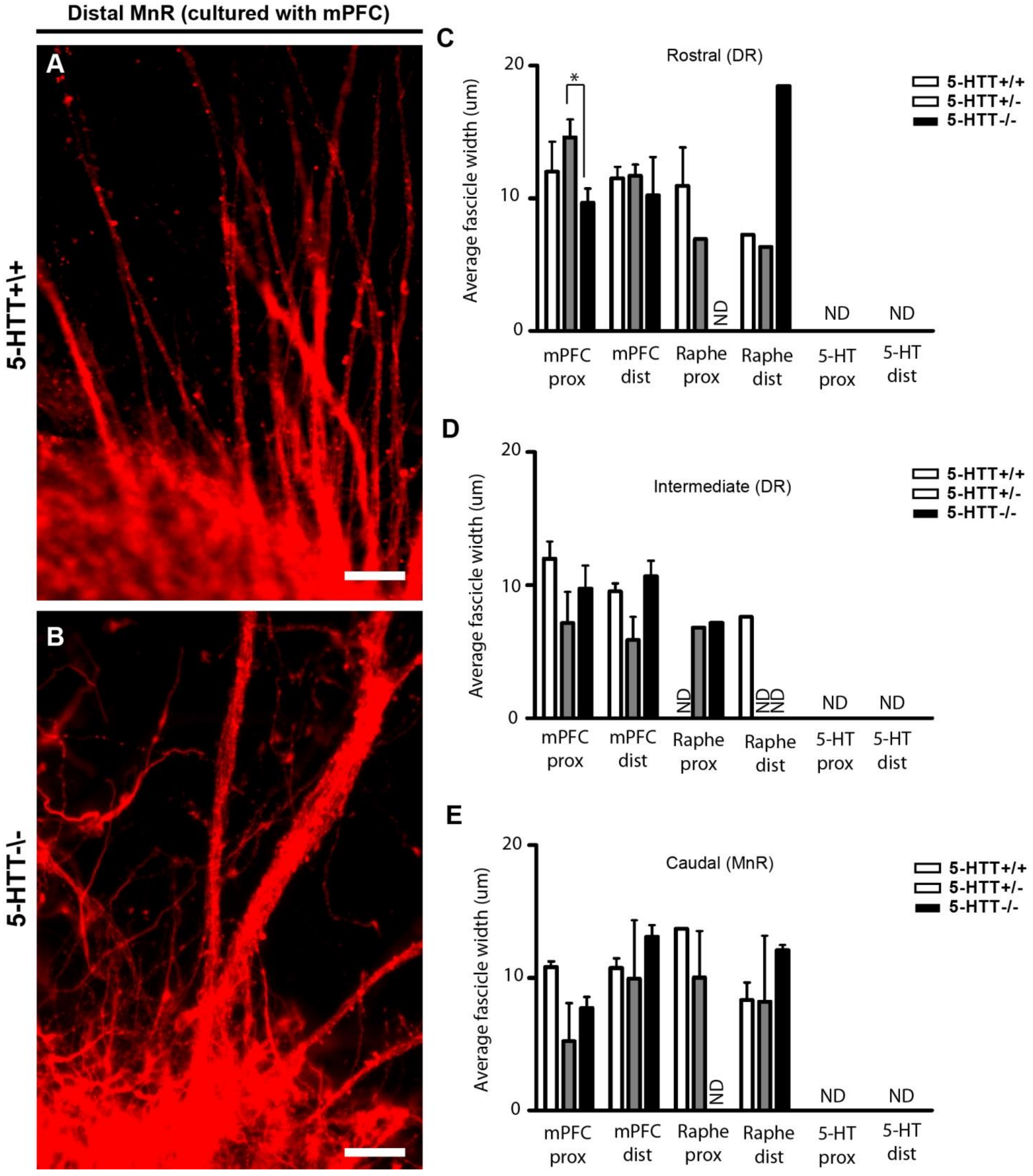

Witteveen et al., Supplemental Figure 4

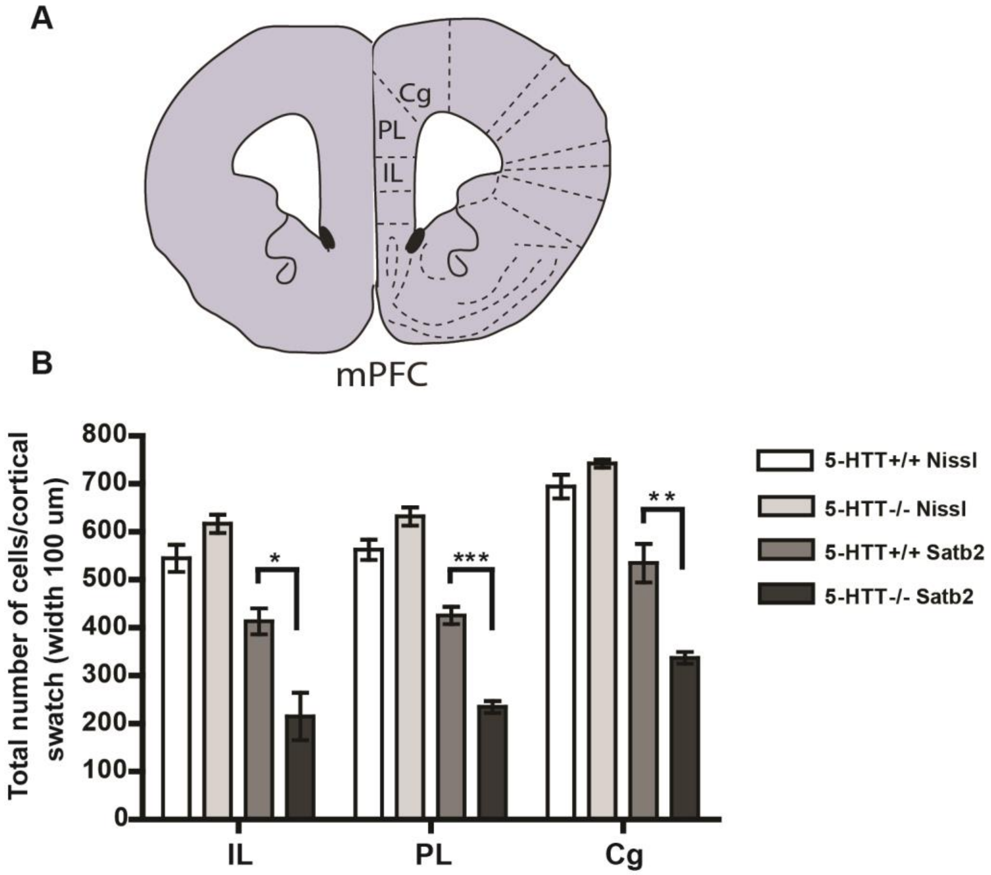

Supplement: Supplemental Figure 1 — Explant assays as a tool to study raphe-prefrontal network formation in vitro. (A) Schematic representation showing the explant microdissection of the subareas of the rostral raphe cluster and the mPFC. The rostral raphe cluster was divided in a rostral (R), intermediate (I) and caudal (C) subarea and duplicated across the midline. Explants in a collagen hill were co-cultured at approximately 300 μm distance from each other. (B) Dorsal view of the dorsal raphe (DR) in a large explant showing 5-HT-positive neurons (green). (B') Enlargement of the boxed area in (B) showing the midline (dashed line) and individual 5-HT neurons sending out their projections. (C) Coronal cryosection showing 5-HT-positive neurons (green) in both the DR and MnR costained with Satb2 (red) and counterstained with fluorescent Nissl (blue). (D) DR explant stained for 5-HT (green) and Tuj1 (red) showing healthy axonal growth cones (asterisk and arrowheads). [file Presentation1.PDF]
